# Supramolecular Chiral Assembly Films with Dynamic Handedness and Emitting-Color Afterglow

**DOI:** 10.1021/acscentsci.5c00847

**Published:** 2025-07-03

**Authors:** Xinkun Ma, Wei Yuan, Wangjian Fang, Letian Chen, Zujin Zhao, Yanli Zhao

**Affiliations:** † School of Chemistry, Chemical Engineering and Biotechnology, 54761 Nanyang Technological University, 21 Nanyang Link, Singapore 637371, Singapore; ‡ State Key Laboratory of Luminescent Materials and Devices, Guangdong Provincial Key Laboratory of Luminescence from Molecular Aggregates, 26467 South China University of Technology, 381 Wushan Rd, Guangzhou 510640, China

## Abstract

This work presents a strategy for the fabrication of multicolor-emitting circularly polarized afterglow (CPA) films by assembling achiral phosphorescent donors and fluorescent emitters in a photonic crystal matrix. Achiral positively charged phosphors and fluorophores with good spectral overlap are selected as the donor and sequential receptors, which are then coassembled with poly­(vinyl alcohol) and cellulose nanocrystals. CPA can be achieved in the blue to near-infrared (NIR) range with stepwise amplification of the dissymmetry factor (lifetime up to 4.52 s and dissymmetry factor up to −0.038) through synergistic chirality and energy transfer processes. Notably, the handedness of the CPA signal can be amplified or reversed by altering the receptor types or the direction of incident light. With the flexibility in manipulating emission and handedness, these materials show potential applications in multidimensional anticounterfeiting and high-definition noctilucent displays.

## Graphical abstract

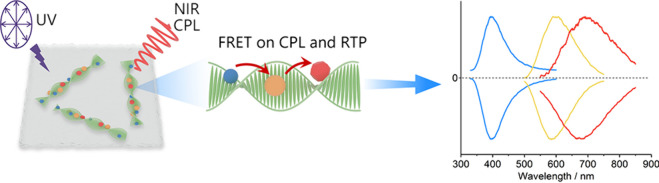

## Introduction

Organic materials that exhibit circularly polarized luminescence (CPL) are of considerable interest owing to their potential applications in anticounterfeiting, 3D displays, and asymmetric catalysis.^1–5^ Various strategies have been developed to prepare CPL-active materials,^6,7^ including the design of chirality-containing chromophores and the assembly of nonchiral chromophores and chiral matrices.^8–10^ Cellulose nanocrystals (CNCs) as a chiral template allow producing highly active CPL when doped with luminophores.^11–16^ However, most reported organic CPL materials rely on fluorescence with fixed emission color and handedness, and circularly polarized afterglow (CPA) materials (with lifetimes greater than 0.1 s) with dynamic emission colors and bidirectional circular polarization remain underexplored.^17–20^ Förster resonance energy transfer based on CPL or room-temperature phosphorescence (RTP) provides a reliable method for increasing emission lifetime and dissymmetry factor (*g*_lum_) as well as expanding the luminescence range.^21–30^ Thus, it was hypothesized that supramolecular chiral assembly resulting from the synergy of long-lived phosphorescent donors and suitable fluorescent acceptors in the chiral template is a facile approach to achieving a dynamic optical functionality for CPA.

In this work, we selected achiral, positively charged phosphors and fluorophores with good spectral overlap as the donor and sequential receptors, which were then coassembled with poly­(vinyl alcohol) (PVA) and CNCs (Scheme 1a). Interestingly, the handedness of the CPA could be easily inverted by controlling the incident light direction, which is attributed to the selective reflection discrepancy arising from the differential CNCs content between the two film surfaces. Furthermore, CPA was expanded from the blue to near-infrared (NIR) range with stepwise amplification of *g*_lum_. Specifically, the triphenylene derivative (TPY) donor with ultralong circularly polarized phosphorescence (lifetime up to 4.52 s, *g*_lum_ up to −0.014, and Φ up to 29%), along with a Förster resonance energy transfer (FRET) process facilitated by the intermediate acceptor rhodamine B (RhB) and the terminal acceptor Nile blue (Nile), exhibited highly efficient and persistent NIR CPL with a lifetime of 2.31 s and a *g*_lum_ value of −0.038 (Scheme 1b,c). These properties are among the best results achieved with NIR CPL systems (Figure S1 and Table S1). Apart from FRET-induced CPL enhancement, the generation and amplification of CPL might arise from the synergy effect of template chirality transfer and the spectral overlap between the photonic bandgaps (PBG) and chromophores luminescence. Finally, the films were successfully applied for multidimensional anticounterfeiting and noctilucent displays.

**Scheme 1:**
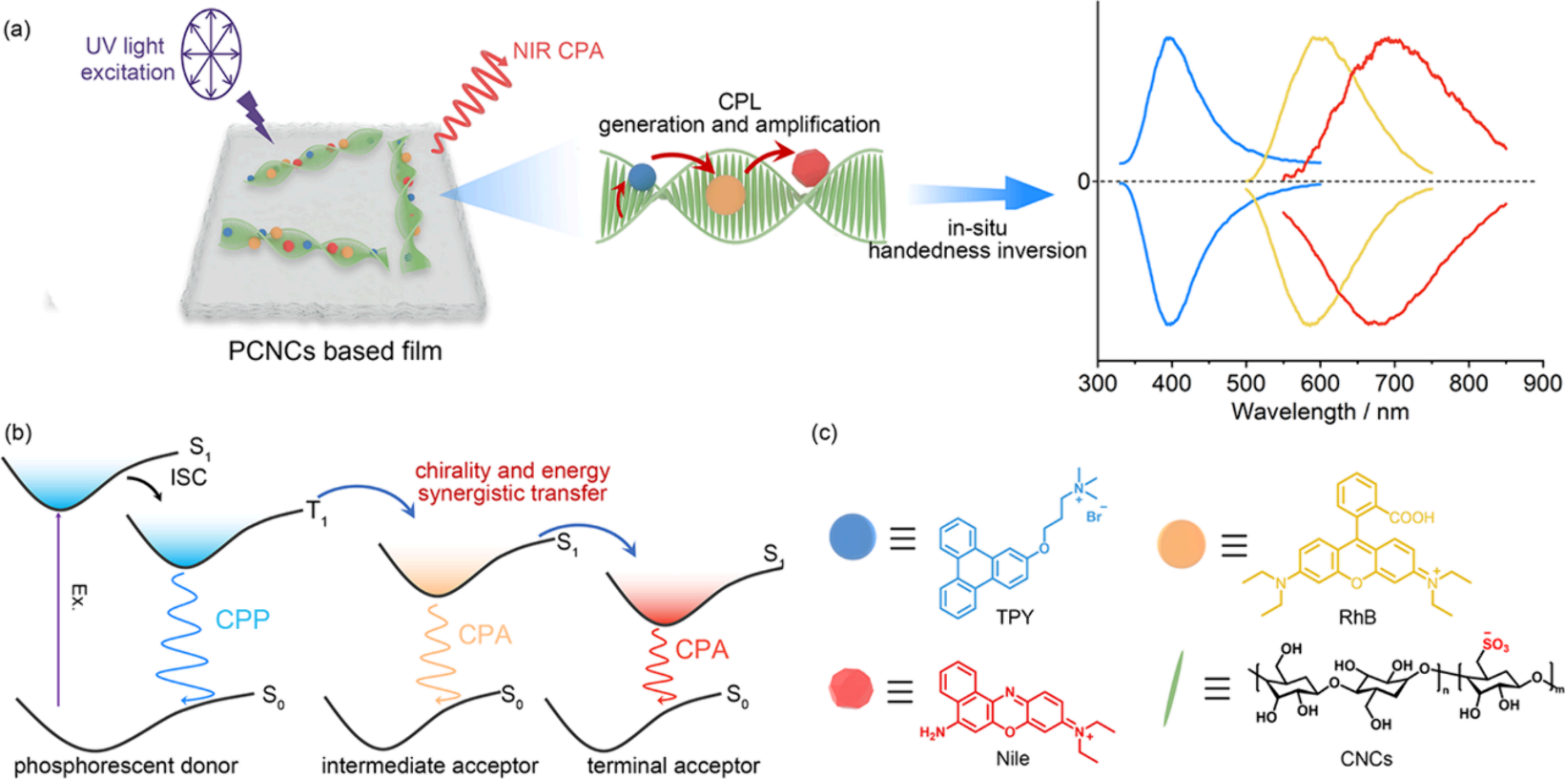
(a) Schematic Illustration for the Achievement of Dynamic CPA through Phosphorescence Energy Transfer and Chirality Transfer, (b) Mechanism of Multicolor-Emitting CPA,^a^ and (c) Chemical Structures of TPY, RhB, Nile, and CNCs

a S_0_, S_1_, and T_1_ refer to the ground state, lowest singlet excited state, and lowest triplet excited state, respectively. Abbreviations: Ex., excitation; ISC, intersystem crossing; CPP, circularly polarized phosphorescence; CPA, circularly polarized afterglow; CPL: circularly polarized luminescence.

## Results and Discussion

The structure and synthetic route of TPY and CNCs are described in Scheme S1. TPY was characterized by high-resolution mass spectrometry and NMR spectra; meanwhile, the purity was confirmed by high-performance liquid chromatography spectra. Stable CNCs suspensions were prepared by sulfating cellulose, and zeta potential analysis and dynamic light scattering (DLS) were performed for characterization (Figure S2). The CNCs films fabricated by evaporative self-assembly were transparent films with excellent irradiance (Figure S2). Scanning electron microscopy (SEM) images suggested that the CNCs were layered and stacked to form a film of approximately 100 μm (Figure S2). Powder X-ray diffraction (XRD) patterns indicated that the CNCs possessed a regular structure (Figure S3).^31^ The circular dichroism (CD) spectra revealed left-handed helical order (Figure 1a and Figure S4). Thus, the zeta potential, DLS, XRD, SEM, and CD results demonstrated that the CNCs were chiral matrices with negatively charged surfaces.

**Figure 1:**
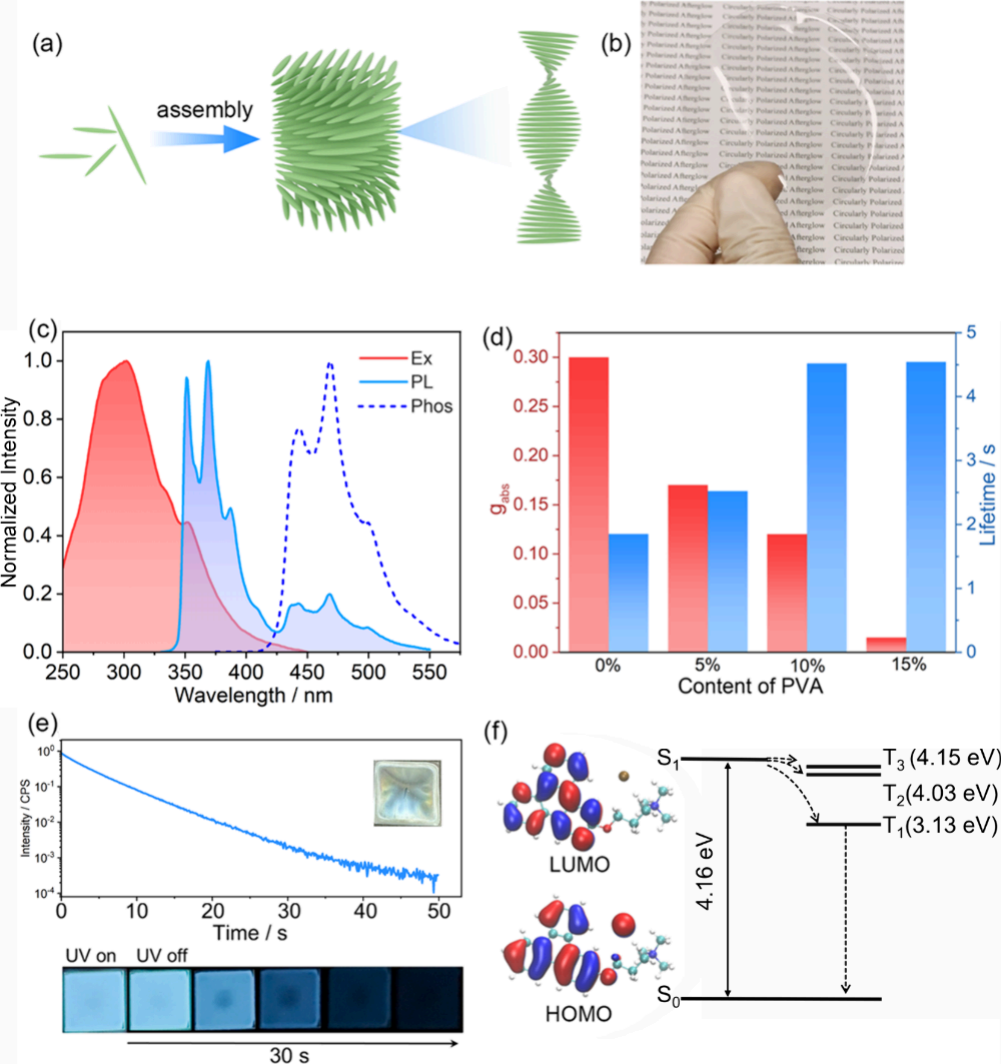
(a) Schematic illustration for the chiral self-assembly process of CNCs. (b) Photographs of large-area TPY-CNCs films (TPY:CNCs:PVA was 0.1:100:15). (c) Excitation spectra, PL spectra, and phosphorescence spectra of TPY-PVA under ambient conditions (λ_ex_ = 300 nm, λ_em_ = 465 nm, time delay 10 ms). (d) Lifetime (blue bars) and *g*_abs_ (red bars) of TPY-PCNCs films with different PVA contents under ambient conditions. (e) (top) Time-resolved decay spectra of TPY-PCNCs films. The inset shows photographs of the TPY-PCNCs film (the mass ratio of TPY:CNCs:PVA was 0.1:100:10, λ_ex_ = 300 nm, λ_em_ = 465 nm). (bottom). Luminescence images of the TPY-PCNCs films before and after the irradiation with 365 nm UV light ended. (f) DFT (M062X/6-311G­(d,p)) calculated frontier molecular orbitals (left) and energy level diagrams (right) of TPY.

Then, TPY was doped into PVA and fabricated into TPY/PVA films to investigate the phosphorescence properties of the isolated phosphors. The CNCs film doped with TPY is maintained as a transparent and rigid film (Figure 1b). The photoluminescence (PL) spectra of TPY/PVA exhibited two emission peaks at approximately 365 and 465 nm (Figure 1c). The nanosecond-scale lifetimes indicated that the 365 nm band could be attributed to fluorescence, whereas the gated emission spectra and second-scale lifetime at 465 nm indicated that the blue band could be attributed to phosphorescence (Figures S5 and S6). Furthermore, a CNCs solution was added with TPY to form transparent films, and the TPY/PVA and TPY-CNCs films displayed similar PL spectra, suggesting that the phosphorescence of the TPY-CNCs film originated from the isolated TPY molecule (Figure S7). To further improve the phosphorescence properties, PVA was coassembled with a TPY-CNCs solution (named TPY-PCNC). The addition of PVA resulted in a significant increase in emission intensity (Φ_phos_ from 1% to 7%) and the lifetime (1.85 to 4.54 s) (Figures S8 and S9 as well as Table S2). However, the PVA content affected the chiral stacking of CNCs (Figure 1d), and the absorptive dissymmetry factor (*g*_abs_) decreased from 0.30 to 0.005 when the PVA content was increased to 15.0 wt %. As seen from the *g*_abs_ and lifetime results obtained at different PVA contents, PCNCs with 10.0 wt % PVA were selected for use as the chiral matrix (Figure 1e). Subsequently, to verify the appropriate phosphorescent donor concentration, TPY-PCNC films with different TPY contents were fabricated, and 0.1 wt % TPY was selected as the donor concentration because low-concentration TPY films exhibited strong phosphorescence emission (τ_phos_ = 4.52 s, Φ = 29%) and avoided destruction of the chiral environment due to subsequent multicomponent assembly (Figures S10 and S11).

Long-lived and effective phosphorescence requires efficient intersystem crossing (ISC) and slow nonradiative decay.^32^ To determine the mechanism of phosphorescence, the optimized structure and frontier molecular orbitals were calculated (Figure 1f). Both the HOMO and LUMO were distributed on the aromatic rings but differed in that the HOMO was spread over an oxygen atom, which implied the possibility of an intramolecular charge transfer. Moreover, modification of positively charged alkyl chains contributed little to the luminescence process. Furthermore, TPY had a small energy gap between S_1_ and T_*n*_, and there were multiple possible transition pathways. The results of theoretical calculations indicated high-efficiency ISC for the populated triplet excitons in TPY. Furthermore, to achieve ultralong RTP, a rigid environment was necessary to suppress nonradiative transitions. For the TPY-PCNCs films, with the addition of PVA, the phosphorescence lifetime (from 1.85 to 4.54 s) and the phosphorescence quantum yield (from 1% to 7%) were improved, which should be attributed to the hydrogen bonding networks and the good TPY dispersion. In the FTIR spectra (Figure S12), the peak corresponding to the hydroxyl group exhibited a red shift and broadening after the addition of PVA, indicating that the PVA enhanced the hydroxyl group bonding to form a denser and more rigid network. In addition, the polymer matrix prevents TPY aggregation. SEM and theoretical calculations were applied to clarify the interaction between TPY and PVA. In SEM images (Figure S13), as the PVA content increases, TPY aggregates become reduced and nearly disappeared at 97 wt % PVA, indicating improved dispersion of TPY within the matrix. The theoretical calculation results revealed the formation of specific hydrogen bonds and C–H···π interactions between TPY and PVA, which contribute to the dispersion of TPY in polymer films (Figure S14). Therefore, PVA serves two synergistic roles in our system: (1) the hydroxyl groups form hydrogen bonds with both CNCs and TPY, immobilizing chromophores to suppress nonradiative decay, and (2) steric hindrance from the PVA chains prevents chromophore aggregation.

Given their excellent RTP performance and negatively charged layer surfaces, TPY-PCNCs films have the potential to serve as platforms for loading suitable dye molecules (Figure 2a). To achieve effective CPA energy transfer, RhB was selected as a first-step triplet energy acceptor because its absorption spectrum overlaps well with the phosphorescence spectrum of TPY (Figure 2b and Figure S15). Triplet-singlet (T-S) FRET between TPY and RhB was observed. As the proportion of energy receptor dye increased, TPY-PCNCs/RhB exhibited a change in its emission color ranging from blue to orange (Figure S16). Moreover, the intensity of the donor phosphorescence peak decreased, and the intensity of the acceptor delayed fluorescence peak increased in the delayed spectra, indicating the occurrence of a high-efficiency energy transfer from the donor to the acceptor (Figure 2c). Impressively, when the mass ratio of the donor to the acceptor was 100:80, the TPY-PCNCs/RhB film exhibited an orange afterglow (τ = 3.27 s, Φ_ET_ = 41%) upon removal of the excitation light (Figure 2d,e and Movie S1). The success of the first step of energy transfer motivated us to construct a system for the second energy transfer step. Nile was selected as the second energy transfer acceptor, because its absorption spectrum overlapped well with the PL spectrum of RhB (Figure S17). With the addition of Nile, the NIR emission peak at 680 nm was dramatically enhanced in the PL and delayed spectra and the intensity of the peak attributed to RhB decreased (Figure S18). The emission color changed from orange to purple. Specifically, when the mass ratio of TPY:RhB:Nile was 100:80:150, TPY-PCNCs/RhB@Nile exhibited persistent NIR luminescence (τ = 2.31 s; Φ_ET_ = 96%) (Figure 2f and Movie S2). The energy transfer efficiency of the second step of energy transfer was greater with Nile than with RhB, possibly because of the different degrees of spectral overlap.

**Figure 2:**
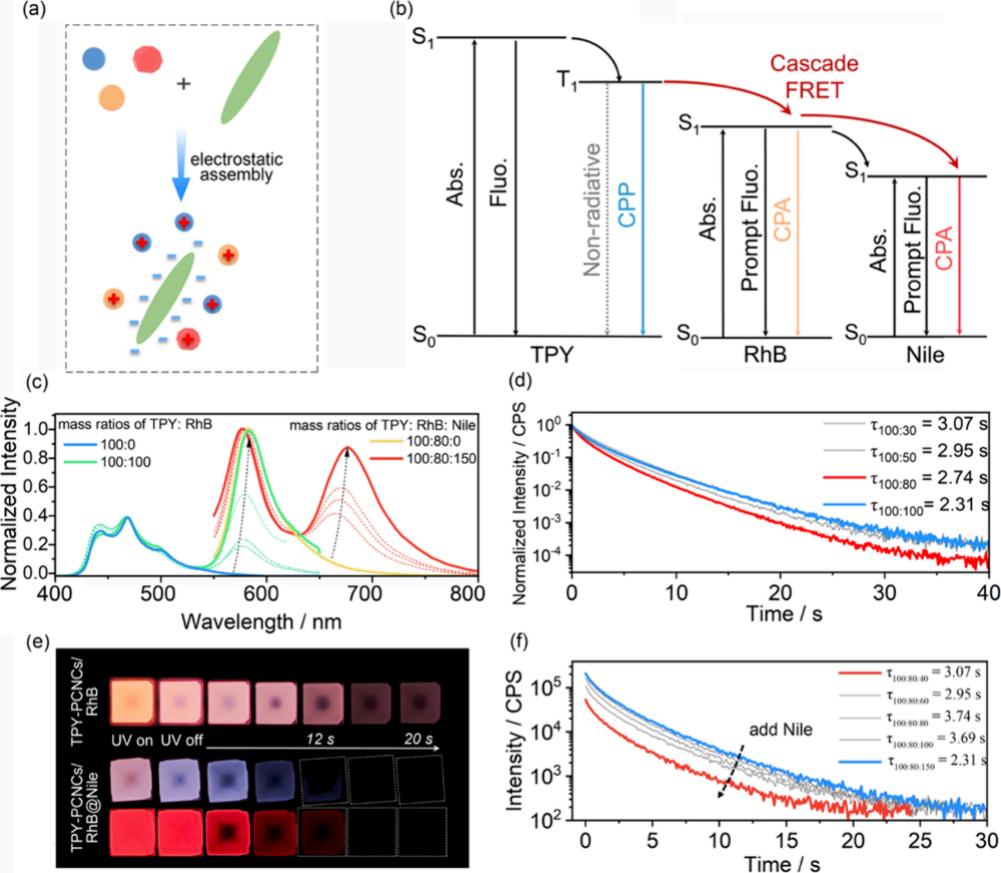
(a) Schematic illustration of the assembly process between CNCs and chromophores. (b) Simplified Jablonski diagram for multicolor-emitting CPA and FRET processes. Abbreviations: ISC, intersystem crossing; Abs., absorption; Fluo., fluorescence; Phos., phosphorescence; PRET, phosphorescence energy transfer. (c) Delayed emission spectra of TPY-PCNCs films with different acceptor concentrations (the green line represents the addition of RhB to the TPY-PCNCs films at mass ratios ranging from 100:0 to 100:100; the red line represents the addition of Nile to TPY-PCNCs/RhB at mass ratios ranging from 100:80:0 to 100:80:150; λ_ex_ = 300 nm, time delay 10 ms). (d) Time-resolved decay spectra of TPY-PCNCs films with different RhB contents (λ_ex_ = 300 nm, λ_em_= 580 nm). (e) Afterglow images of TPY-PCNCs films doped with different acceptors under ambient conditions (top, TPY-PCNCs/RhB films, *m*_TPY_:*m*_RhB_ = 100:80; middle: TPY-PCNCs/RhB@Nile films observed with the naked eye; bottom: PCNCs/RhB@Nile films observed with an NIR filter, 650–2000 nm). (f) Time-resolved decay spectra of TPY-PCNCs/RhB with different Nile contents (λ_ex_ = 300 nm, λ_em_= 680 nm).

To demonstrate that afterglow energy transfer occurred from the triplet state to the singlet state and then to another singlet state, the steady-state and delayed spectra upon assembly with acceptor dyes were measured (Figures S19–S23). The emission peaks for RhB and Nile remained the same in the steady-state and delayed spectra, implying that the acceptors underwent only radiative transitions from singlet excitons during the energy transfer process. The possibility of singlet-singlet (S-S) FRET between TPY and RhB was further examined. Fluorescence lifetimes were measured upon the addition of different amounts of RhB to the TPY-PCNCs films, and the lifetimes at 390 and 580 nm were not altered but were similar to those of dyes doped with PVA alone (), which indicated that no S-S energy transfer process occurred during the first step of the FRET process. With further addition of Nile, the nanosecond-scale lifetime at 580 nm decreased as did that at 680 nm. That is, there are two approaches to S-S FRET between RhB and Nile, which were prompt fluorescence energy transfer and delayed fluorescence energy transfer. In summary, all of the donors and acceptors absorbed the energy of the excitation light, and then the radiative transition of TPY included fluorescence and phosphorescence. The radiation transitions of the acceptor dyes involved only prompt fluorescence, and direct S-S FRET occurred between the two receptors. In addition, the most notable process was T-S-S FRET, in which the long-lived TPY triplet exciton energy was directly transferred to the singlet state of the acceptor, resulting in a long-lived delayed fluorescence.

The chiral transfer process was then investigated. The chirality of the assembly was confirmed through CD and powder XRD measurements. For TPY-PCNCs, TPY-PCNCs/RhB, and TPY-PCNCs/RhB@Nile (Figure 3a), the strong positive Cotton effect indicated that the left-handed helical structure was retained after the assembly (|*g*_CD_| values for TPY-PCNCs, TPY-PCNCs/RhB, and TPY-PCNCs/RhB@Nile are 0.10, 0.11, and 0.04, respectively). The strong signals at approximately 650 nm were assigned to the PBG from the CNCs, suggesting that the assembly of trace amounts of chromophores did not affect the ordered stacking of the CNCs. Interestingly, peaks attributed to TPY, RhB, and Nile appeared in the CD spectra. This observation suggested that, upon assembling with PCNCs, chirality was transferred from CNCs to chromophores, which might be attributed to supramolecular electrostatic assembly (Figure 3b). Powder XRD provided additional evidence as strong CNCs diffraction peaks were detected for both the pure CNCs films and the multicomponent-assembled PCNCs films, implying that the chiral environment was preserved (Figure S28).

**Figure 3:**
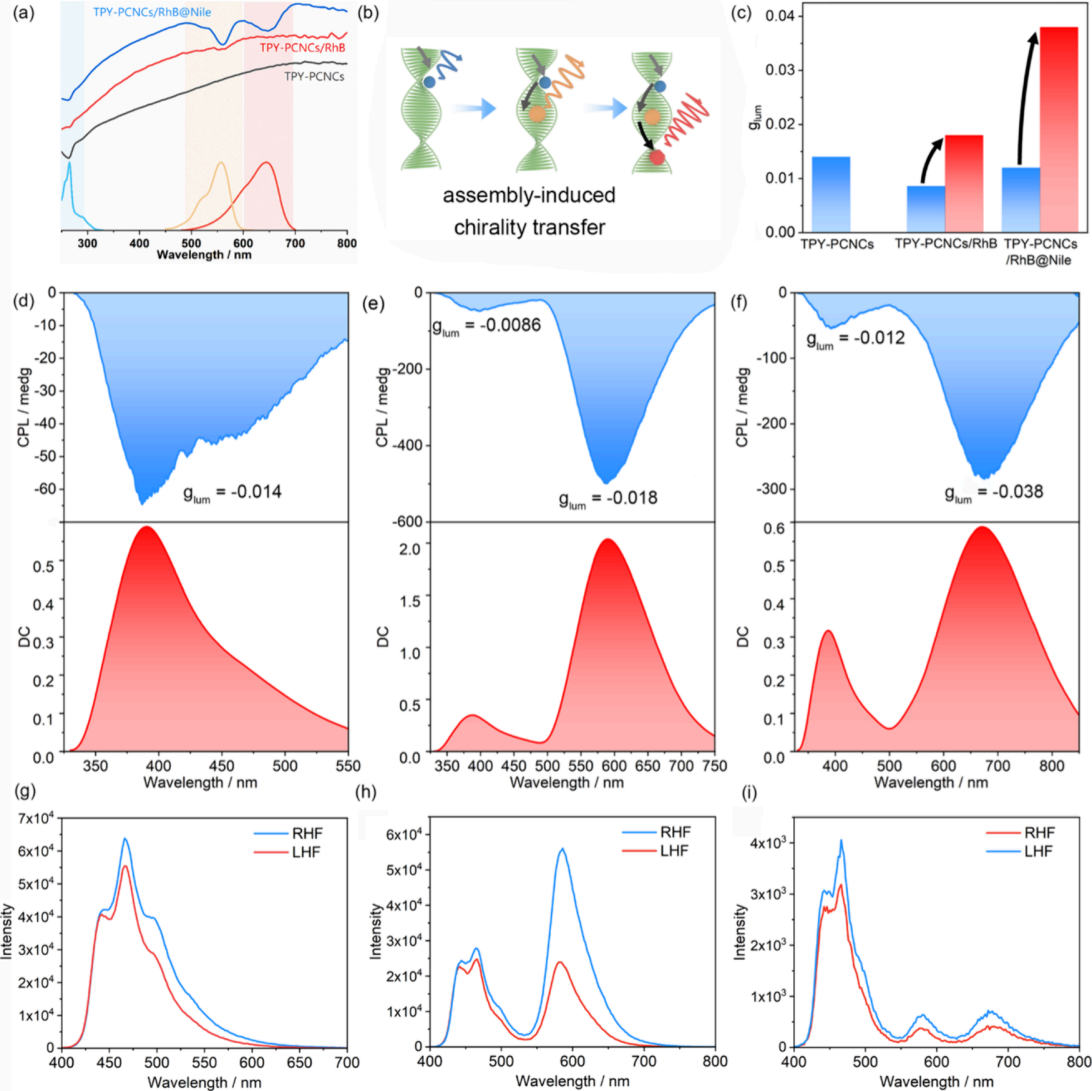
(a) CD spectra (top) of TPY-PCNCs, TPY-PCNCs/RhB, and TPY-PCNCs/RhB@Nile film and UV–vis absorption spectra (bottom) of TPY-PVA, RhB-PVA and NilePVA. (b) Schematic illustration for the generation and amplification of chirality. (c) *g*_lum_ values of TPY-PCNCs films (blue bar 475 nm), TPY-PCNCs/RhB films (blue bar 475 nm and red bar 600 nm), and TPY-PCNCs/RhB@Nile films (blue bar 475 nm and red bar 700 nm). (d–f) CPL spectra of the TPY-PCNCs films, TPY-PCNCs/RhB films (D-A mass ratio = 100:80), and TPY-PCNCs/RhB@Nile films (D-A mass ratio = 100:80:150) (λ_ex_ = 300 nm). (g–i) Delayed emission spectra of the TPY-PCNCs, TPY-PCNCs/RhB, and right-handed circularly polarized filter (LHF) and right-handed circularly polarized filter (RHF).

The CPL properties were further evaluated. The CNCs behaved like a polarizer, selectively reflecting left-handedness CPL and transmitting right-handedness CPL, and the wavelength of polarization was related to the PBG wavelength.^12,33^ In our systems, the PBG covered the visible and NIR regions, which provided the preconditions for multicolor-emitting CPL. As shown in Figure 3c, all of the films exhibited strong negative CPL signals with *g*_lum_ values of approximately −1 × 10^–2^. Interestingly, |*g*_lum_| increased with the energy transfer. The |*g*_lum_| values for TPY-PCNCs/RhB were 0.0086 at 390 nm and 0.018 at 580 nm, which represented a 2.1-fold improvement after energy transfer. The |*g*_lum_| values for TPY-PCNCs/RhB@Nile were 0.012 at 390 nm and 0.038 at 680 nm, which represented a 3.2-fold improvement after a two-step energy transfer (Figure 3d–f). To examine the contribution of CNCs as chiral templates to the CPL,^34^ the CPL spectra of the CNCs films assembled with individual chromophores were measured, and similar values of |*g*_lum_| of approximately 0.02 were obtained for all three films (Figure S29). Moreover, the CPL spectra with different excitation wavelengths were measured, and |*g*_lum_| significantly decreased as the excitation wavelength was red-shifted (Figures S30 and S31). This result suggests that the origin of CPL is not only the filtration of CNCs but also from supramolecular chiral assembly. According to reported results, during the transfer of energy and helicity, the helicity from a rotating donor dipole can be conserved, and the transfer of luminescence helices originating from rotating dipoles can be coupled with FRET, showing the potential to increase *g*_lum_ values when a coupling between electric and magnetic transition dipoles is involved during FRET.^25,35^ Since the CPL-300 spectrometer provided only steady-state spectra, to confirm that phosphorescence or delayed fluorescence was circularly polarized, the delayed spectra were acquired on a Horiba FluoroMax-4 spectrofluorometer (equipped with a pulsed-xenon excitation source) through a circularly polarized filter. As shown in Figure 3g–i, the CNCs-based films exhibited differences in their emission intensity under filters with different handedness. For comparison, no CD or CPL was detected for the PVA-based film (Figures S32 and S33).

Notably, handedness inversion of the CPL signal was realized by changing the incident light direction (Figure 4a,b), indicating that the signal was positively or negatively converted by inverting the incident light or films. The spectra before and after the inversion show obvious symmetry; that is, no change in the emission wavelength (Figure 4c). Moreover, the |*g*_lum_| values of the right-handed emission were greater than those of the left-handed emission. In order to gain insight into the handedness inversion mechanism, we first investigated the artifact effect due to linear birefringence effect. We measured the CD spectra when rotating the film sample along the optical axis. As shown in Figures S34–S37, the CD sign value fluctuated around 500 mdeg, suggesting that the observed CD signal represents the authenticity of helical chirality in the CNCs. Meanwhile, the CD signal does not follow the cosine function, which demonstrates that the contribution of LD effects in the strong CD signals can be neglected. Then, the CD spectra for both sides of the films were obtained, and all of the films displayed the same CD signals after being inverted, indicating that the inversion of the films did not lead to the chirality inversion of CNCs (Figure S38). It is well-known that CNCs enable the division of incident light into left-handed and right-handed CPL by selective reflection and transmission dictated by a left-handed helical sense, which reflects left-hand polarized light and transmits right-hand polarized light.^12^ Commonly, the reflected light wavelength of CNCs depends on the incident angle, and the reflection wavelength shifts to shorter wavelengths according to Bragg’s condition at oblique incidence. Then, the CPL spectra with different incident light angles were measured (Figure S39). Signal inversion was activated only when incident light reached a different surface, which suggested that the inversion of the CPL signal cannot be attributed to the incident light angle dependence on the degree of overlap of the PBG with the PL spectra.

**Figure 4:**
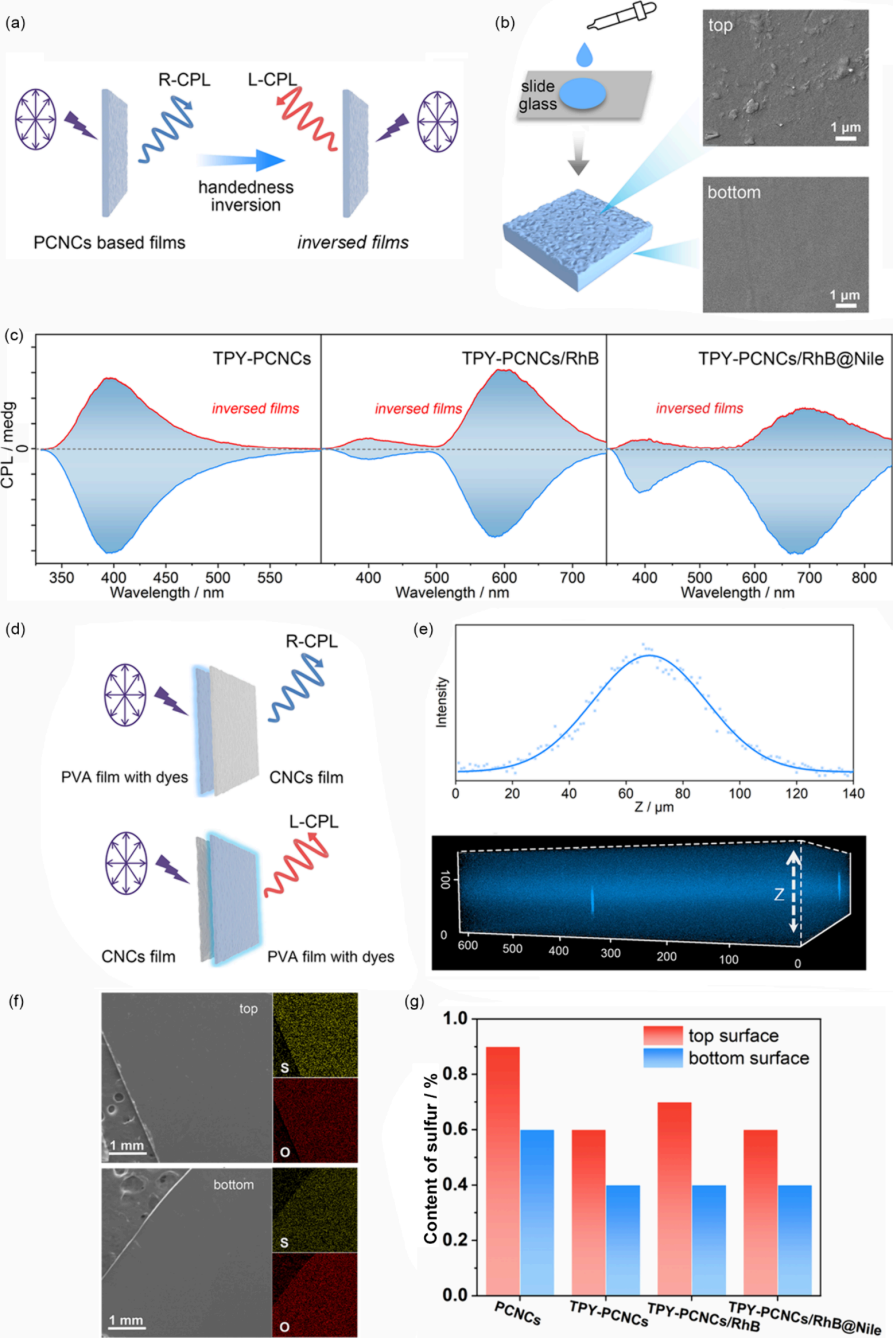
(a) Schematic illustration of CPL handedness inversion by changing the incident light direction. (b) Preparation process of PCNCs-based films. Inset images: (top) SEM image of the top surface of the films; (bottom) SEM image of the bottom surface of the films. (c) CPL spectra before and after reversal of the TPY-PCNCs films (left), TPY-PCNCs/RhB films (middle, D–A mass ratio is 100:80), and TPY-PCNCs/RhB@Nile films (right, D–A mass ratio is 100:80:150) (λ_ex_ = 300 nm). (d) Schematic illustration of handedness inversion by exchanging the positions of CNCs and dye-PVA films. (e) *Z*-axis luminescence spectra of the TPY-PCNCs films (top) and confocal z-stack images of the TPY-PCNCs films (bottom). (f) SEM-EDS mapping imaging of PCNCs (more details are shown in Figure S42). (g) Comparison of sulfur content based on the top and bottom surfaces of PCNCs-based films according to EDS spectroscopy (C, N, O, and S elements were collected).

Thus, we hypothesized that the handedness inversion might be attributed to the different proportions of transmission and reflection. TPY-PVA, RhB-PVA, Nile-PVA, and CNCs films were prepared. Afterward, the CNCs films were placed between the detector and the PVA films, and strong R-CPL emission was detected, which resulted from the selective transmission of R-CPL by the CNCs. Then, the CNCs were placed between the light source and the PVA films, and the signals were reversed, such that L-CPL was detected, which resulted from the selective reflection of L-CPL by the CNCs (Figure 4d and Figure S40). By careful comparison, it was found that the right-handed CPLs generally had higher |*g*_lum_| values than did the left-handed CPLs, which was consistent with the findings for the TPY-PCNCs composite films. In comparison, inverting the CNCs film did not lead to a change in the CPL signal (Figure S41). Thus, the possible explanation for the handedness inversion was the surface distribution differences in the chromophores or CNCs. Confocal microscopy Z-stacking imaging was used to measure the homogeneity of the chromophore in the film. 3D imaging or *Z*-axis luminescence intensity spectra revealed that the chromophores were homogeneously assembled without aggregation (Figure 4e). Thus, handedness inversion might be induced by the inhomogeneous distribution of the CNCs during evaporative self-assembly.

X-ray photoelectron spectroscopy (XPS) and SEM-EDS were applied to check the CNCs distribution and content. From the SEM mapping images, a uniform distribution of sulfur elements on the film surface was observed (Figure 4f). However, the sulfur content of the top interface from the PCNCs-based films was obviously different from that of the bottom interface. The distribution at the top is denser, which provides more evidence of CPL chiral inversion (Figure 4g and Figures S42–S45). XPS studies exhibited similar results (Figure S46). This observation suggests that in the formation of the film, a denser CNCs layer is formed at the top of the film, which is similar to the principle in Figure 4d, where the top CNCs acts as a chiral filter. SEM imaging provided more evidence (Figure 4b): the film side exposed to air was rougher than the side adjacent to the slide. The rougher side allowed for enhanced reflection of light. Thus, when light was incident from the air side, the result was similar to that of “CNCs to dyes/PVA” (bottom of Figure 4d) as in the case of CNCs, resulting in left-handed CPL. When the light was incident from the slide side, the result was similar to those of dyes-PVA-CNCs (top of Figure 4d), resulting in right-handed CPL. Another piece of evidence was that the |*g*_lum_| values of the right-handed CPLs obtained relative to the CNCs used as filters alone were greater than those of the composite film, which suggested that there was competition between the right- and left-handed CPL in the composite film and that the positive and negative |*g*_lum_| values were precisely the result of competition between the reflected and transmitted light.

The supramolecular assembly not only improved the luminescence performance but also enhanced the FRET process. In this system, the proportion of chromophores was trace and approximately 0.1 wt %, and the negatively charged CNCs acted as anchor points to pull the individual cationic components closer together. Compared with Nile, Cy5 has similar absorption and emission spectra but is classified as an anionic fluorophore. After doping with Cy5, in the PL and gated emission spectra, the emission of the RhB donor did not decrease upon the addition of Cy5, nor did it increase the Cy5 emission, which was attributed to inefficient energy transfer due to a lack of assembly (Figure S47).

Figure 5 shows the potential applications of multimode CPL materials. Specifically, depending on the components, the films can exhibit different properties of luminescence. As shown in Figure 5a, TPY-PCNCs were used as inks to print letters. There was almost no display under daylight or UV light, but a clear blue afterglow appeared when the light source was removed (Movie S3). Furthermore, the transparent films were applied for a high-definition noctilucent light source under low-light conditions. As shown in Figure 5b, the TPY-PCNCs films were covered on the paper. After removal of the light source, the naked eye could still capture the “circularly polarized afterglow” words. In addition, given the multicolor-emitting, time-dependent, and ambidextrous CPL, these polymeric materials were applied for multilevel anticounterfeiting and information encryption (Figure 5c). A series of PVA-based films were produced: acceptor-only PVA films, D–A PCNCs films, and D–A films without CNCs. These films were arranged as needed to form the required letters (Movie S4). Under daylight, “97” was identified by the naked eye because the donor-only films were colorless and transparent, while the other films effectively absorbed visible light. Moreover, “471” was observed under a UV lamp, because of its strong luminescence. Once the UV lamp was turned off, the third information, “441”, was clearly revealed. After a short period, the information observed by the naked eye was “411”. The fourth level of information was displayed when the sentence changed to “41”, the fifth level of information was “4”, and the final level of information was “L”. In addition, chirality is another dimension of the information. The observation of only “111” was a result of chiral PL, and CPL-based information was detected using a specific instrument. Consequently, multilevel information encryption and anticounterfeiting were realized via multidimensional luminescence based on absorption, luminescence color, lifetime, and chirality.

**Figure 5:**
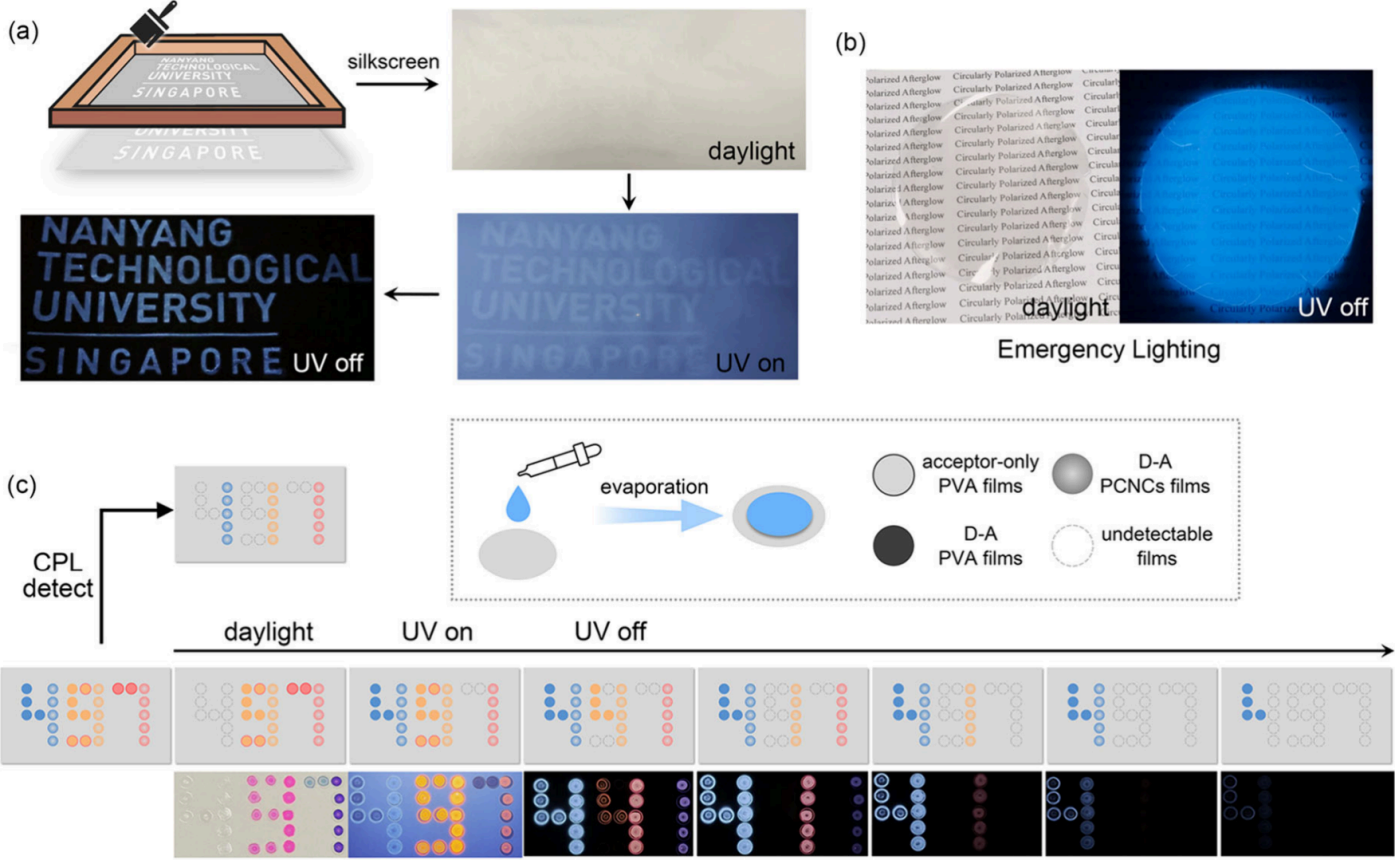
(a) Schematic illustration and photographs of TPY-PCNCs films applied for screen printing. (b) Luminescence photographs of large-area TPY-PCNCs films applied for a noctilucent light source (the mass ratio of TPY:CNCs:PVA was 0.1:100:15). (c) Schematic illustration of the application process for multilevel anticounterfeiting and information encryption. The blue dots are TPY-based films, the orange dots are RhB-based films, the red dots are Nile-based films, *m*_TPY_:*m*_CNCs_:*m*_PVA_ = 0.1:100:10, [TPY]:[RhB] = 100:80, [TPY]:[RhB]:[Nile] = 100:80:150, the excitation light source is a 365 nm hand-held lamp, and the print stock is paper without background fluorescence.

## Conclusion

In summary, a facile strategy has been presented to achieve large-area CPA materials via synchronous phosphorescence and CPL FRET in a CNCs-based polymer matrix. This strategy relies upon supramolecular assembly between a phosphorescence donor, fluorescence acceptor, and chiral matrix, which effectively suppresses the nonradiative decay and promotes triplet-to-singlet energy transfer and chirality transfer processes. With the advantages of tunable emission colors, lifetimes, and handedness, these films show a promising potential for uses in multifunctional applications. This work not only provides a versatile strategy for developing CPL-active multicolor-emitting afterglow materials but also opens an avenue to expand the features of organic afterglow.

## Experimental Section

### Reagents and Measurements

All reagents and solvents were purchased from commercialized suppliers and used as supplied except for the use of other reagents, unless otherwise noted. ^1^H NMR (400 MHz) and ^13^C NMR (100 MHz) spectra were obtained with a Bruker BBFO 400 Spectrometer. UV–vis absorption spectra were recorded on a Shimadzu UV-3600 spectrophotometer. HPLC studies were performed on a Shimadzu LC-20AR instrument equipped with an XB-C18 column. Photoluminescence (PL) spectra, time-correlated decay profiles, and quantum efficiency were processed on a Horiba Fluoromax-4 spectrofluorometer or an Edinburg FLS-1000 fluorescence spectrometer. Powder X-ray diffraction (XRD) patterns were obtained at a Rigaku SmartLab 3K equipment. X-ray photoelectron spectroscopy (XPS) experiments were carried out on Kratos AXIS Supra^+^. Scanning electron microscopy (SEM) images and SEM-EDS were carried on an JSM-7600F Schottky Field Emission Scanning Electron Microscope. Circular dichroism (CD) measurements were performed on a Jasco J-1500 circular dichroism spectrometer equipped with a PFD-425*S*/15 Peltier-type. Circularly polarized luminescence (CPL) measurements were performed with a JASCO CPL-300 spectrometer or Horiba Fluoromax-4 spectrofluorometer equipped with a circularly polarized filter. The DC value in the bottom of the CPL spectrum stands for fluorescence intensity. A 0.1 mm optical range solid powder was used for measuring the CD and CPL spectra with transmittance mode. For CD and CPL testing, the film sample is aligned perpendicular to the incident light unless otherwise noted. To estimate the contribution of the LD effect on the true CD signal, CD was measured in steps of 45° by rotating the sample. To estimate the contribution of PBG on the true CPL signal, CPL with different angles of incidence was measured by rotating the sample.

### Confocal Microscopy Z-Stacking Imaging

The polymeric films were used directly for confocal imaging with the image Z-Stack mode. The number of slices or images taken through the structure can be manually chosen depending on the definition desired. Step size is the distance in 1 μm that the objective would move on the *Z*-axis between each captured image. After the Z-stacked image is captured, a 3D image can be obtained from the stacked image. *Z*-axis luminescence spectra are obtained by averaging the luminous intensity of single z-plane images.

### Energy-Transfer Efficiency (Φ_ET_)

The fraction of the absorbed energy that is transferred to the acceptor is experimentally measured as a ratio of the phosphorescence intensities of the donor in the absence and presence of the acceptor (*I*_D_ and *I*_DA_).^36^

$$\Phi_{ET}=1-\frac{I_{DA}}{I_{D}}$$

### Synthesis of Compound **2**

Compound **1** (1.00 g, 4.1 mmol) and 1,3-dibromopropane (1.6 g, 8.2 mmol) were dissolved in DMF (10 mL), which were then heated at 100 °C overnight. The crude product was dissolved in CH_2_Cl_2_ and was extracted three times with water. The organic layer was dried with anhydrous Na_2_SO_4_ and filtered, and the solvent was removed by evaporation. The resulting residue was further purified by column chromatography (hexane/ethyl acetate). Compound **2** was obtained as a white solid (0.90 g, 60%). ^1^H NMR (400 MHz, CD_3_Cl): δ 8.67 (m, 2H), 8.59 (d, *J* = 6.9 Hz, 3H), 8.10 (s, 1H), 7.67 (m, 4H), 7.31 (m, 1H), 4.38 (t, *J* = 5.8 Hz, 2H), 3.73 (t, *J* = 6.3 Hz, 2H), 2.47 (t, *J* = 6.1 Hz, 2H). ^13^C NMR (100 MHz, CD_3_Cl): δ 158.08, 131.29, 130.22, 129.87, 129.44, 128.81, 127.37, 127.28, 127.06, 126.33, 124.95, 123.98, 123.98, 123.39, 123.25, 123.29, 122.80, 106.68, 65.57, 32.50, 30.10. HRMS (ESI): *m*/*z* for C_27_H_28_Br_3_N_3_ calcd [M + H]^+^ 365.0463, found 365.0460.

### Synthesis of TPY

Compound **2** (1.00 g, 2.8 mmol) was dissolved in trimethylamine (20 mL, ca. 13% in tetrahydrofuran) and stirred overnight. The precipitate was collected by filtration and washed several times with tetrahydrofuran. TPY was obtained as a white solid after being dried (0.94 g, 81%). ^1^H NMR (400 MHz, DMSO-*d*_6_): δ 8.77 (m, 5H), 8.24 (s, 1H), 7.71 (m, 4H), 7.36 (d, *J* = 8.6 Hz, 1H), 4.37 (t, *J* = 6.0 Hz, 2H), 3.66–3.55 (m, 2H), 3.16 (s, 9H), 2.31 (m, 2H). ^13^C NMR (101 MHz, DMSO-*d*_6_): δ 158.28, 131.27, 130.04, 129.75, 129.33, 128.63, 128.26, 128.12, 127.91, 127.16, 125.91, 124.41, 124.07, 124.01, 123.78, 123.55, 117.25, 107.20, 65.62, 63.50, 52.84, 52.80, 52.77, 23.17. HRMS (ESI): *m*/*z* for C_27_H_28_Br_3_N_3_ calcd. [M – Br]^+^ 344.2014, found 344.2021.

### Preparation of CNCs

CNCs were synthesized according to the literature.^37^ Typically, cellulose powder (20 g) and H_2_SO_4_ aqueous solution (175 mL, 64 wt %) were mixed under vigorous stirring at 45 °C for 1 h. Then, the mixture was diluted with cold deionized water (2000 mL) to stop the hydrolysis and allowed to settle overnight. The clear top layer was decanted, a cloudy suspension was obtained after centrifugation, and this process was repeated three times to remove soluble cellulose. Then, the suspension was placed inside dialysis membrane tubes (12000 molecular weight cutoff) and dialyzed against water for 5 days. The final product was a white suspension, and the content of CNCs was determined by taking a small amount of the solution and measuring the mass before and after drying.

### General Preparation for the CNCs Film

After ultrasonic treatment for 20 min, the CNCs solution (1.0 mL, 3.0 wt %) was dropped on a slide and then placed in a glass desiccator containing phosphorus pentoxide desiccant. After 48 h of desiccation, CNCs films were obtained.

### General Preparation for the PCNCs Film

After ultrasonic treatment for 20 min, a quantitative amount of PVA solution (50 mg/mL) was added to the CNCs solution (1.0 mL, 3.0 wt %), which was then magnetically stirred for 10 min. After settling until no bubbles were generated in the solution, the mixed solution (1.0 mL) was deposited dropwise onto the glass slide surface, which was then transferred to a glass desiccator containing phosphorus pentoxide. After 48 h of desiccation, freestanding PCNCs were fabricated.

### General Preparation for the PCNCs Film with Donor or D–A Dyes

After ultrasonic treatment for 20 min, a quantitative amount of dye solution ([TPY] = [RhB] = [Nile] = 1.0 mg/mL) was added to the CNCs solution (1.0 mL, 3.0 wt %). Then, a PVA solution (50 mg/mL) was added to the mixed solution, which was magnetically stirred for 10 min. After settling until no bubble was generated in the solution, the mixed solution was added dropwise to the surface of the slide and then placed in a glass drying dish containing phosphorus pentoxide desiccant. After drying for about 2 days, the TPY-PCNCs, TPY-PCNCs/RhB, or TPY-PCNCs/RhB@Nile films were obtained. They were dried in an oven at 60 °C for 2 h to remove water for spectral measurements.

### Theoretical Calculations

Molecular geometry optimizations were performed for the ground state at the level of M062X/6-311G­(d,p). The excitation energies were calculated by using TDDFT for electronic excited singlet and triplet states at the level of M062X/6-311G­(d,p). To analyze the noncovalent interactions, the Independent Gradient Model based on Hirshfeld partition (IGMH) method was employed.^38^ This analysis was performed with the Multiwfn 3.8 (dev) program, utilizing the wave functions from the optimized structures.^39^ The resulting IGMH isosurfaces were rendered using the Visual Molecular Dynamics (VMD) software.^40^

## Supplementary Material











## Notes

Synthetic route, HPLC spectra, DLS curves,zeta potential, SEM images, powder XRD patterns, UV–vis absorption and CD spectra, time-correlated decay curves, PL spectra, FTIR spectra, phosphorescence spectra, gated emission spectra, CPL spectra, and NMR spectra (PDF)
Afterglow of TPY-PCNCs, TPY-PCNCs/RhB or TPY-PCNCs/RhB@Nile films with hand-held UV lamp (MOV)
Persistent NIR luminescence of TPY-PCNCs/RhB@Nile film with NIR filter (MOV)
TPY-PCNCs used as an ink to print letters (MOV)
Multilevel anticounterfeiting and information encryption using different PCNCs-based films (MOV)
