# Supplementary material for: Supramolecular Chiral Assembly Films with Dynamic Handedness and Emitting-Color Afterglow
Source: ACS Cent Sci. 2025 Jul 3;11(7):1230–9. doi: 10.1021/acscentsci.5c00847 (PMC12291125; doi:10.1021/acscentsci.5c00847)
Supplement: Supplementary file 1 [file oc5c00847_si_001.pdf]

Supporting Information

Supramolecular Chiral Assembly Films with Dynamic Handedness and Emitting-Color Afterglow

Xinkun Ma,[†] Wei Yuan,[†] Wangjian Fang,[†] Letian Chen,[‡] Zujin Zhao*[‡] and Yanli Zhao*[†]

[†]School of Chemistry, Chemical Engineering and Biotechnology, Nanyang Technological University, 21 Nanyang Link, Singapore 637371, Singapore

[‡]State Key Laboratory of Luminescent Materials and Devices, Guangdong Provincial Key Laboratory of Luminescence from Molecular Aggregates, South China University of Technology, 381 Wushan Rd, Guangzhou, Guangzhou, 510640 China

Email: mszjzhao@scut.edu.cn; zhaoyanli@ntu.edu.sg

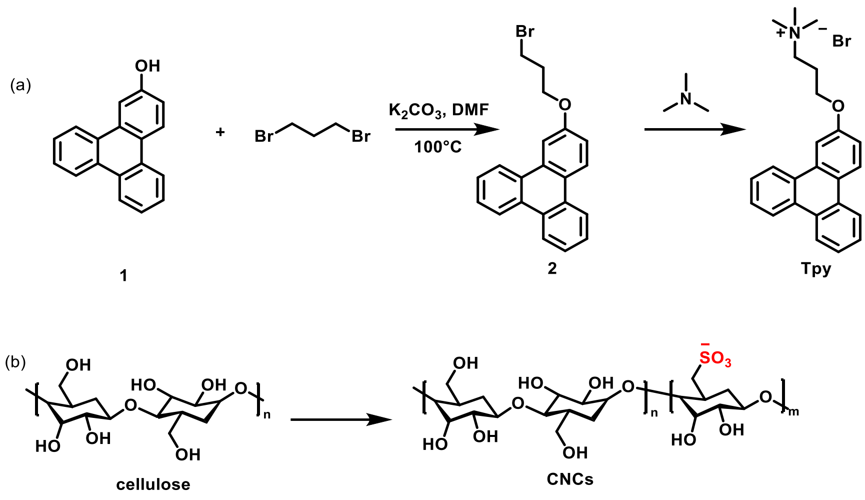

Scheme S1. (a,b) Synthetic route of compounds.

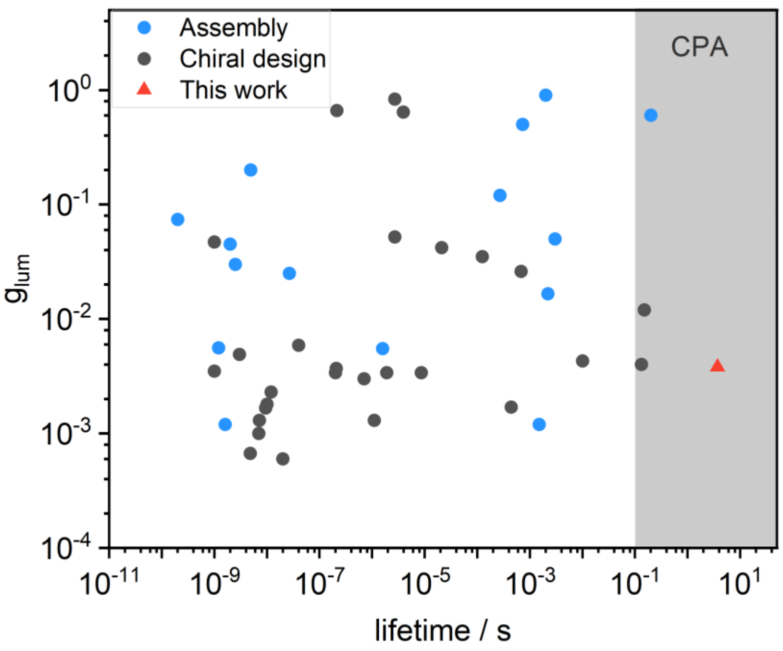

Figure S1. Lifetime and g_{lum} of NIR CPL materials reported in recent years.

Table S1. Lifetime and g_{lum} of NIR CPL materials reported in recent years.

Material name	Source of chirality	g_{lum}	Lifetime	Reference
This work	assembly	0.0038	3.69 s	NA
Metal halide perovskite	assembly	0.9	2.0 ms	*ACS Nano* **2024**, *18*,

nanocrystals				15888–15897
S/R-ONTAP	chiral design	0.0017	9.4 ns	*Adv. Opt. Mater.* **2024**, *12*, 2303155
ITIC	assembly	0.074	0.2 ns	*Adv. Funct. Mater.* **2025**, *35*, 2423077
Yb(III) complexes	chiral design	0.052	2.7 us	*Chem. Eur. J.* **2025**, e202500910
Perovskite	magneto-optical effect	0.0043	10 ms	*Magnetochemistry* **2024**, *10*, 39
Lanthanide-based nanoparticles	assembly	0.5	728 μs	*ACS Nano* **2023**, *17*, 2661–2668
Pyrene/Cy5	assembly	0.025	26.7 ns	*Angew. Chem. Int. Ed.* **2024**, *63*, e202407385
BODIPY derivatives	chiral design	0.0035	1.0 ns	*Aggregate* **2024**, *5*, e498
Quantum dots	chiral design	0.0034	~200 ns	*Nano Today* **2024**, *58*, 102436
CY7-based nanoparticles	assembly	0.0056	1.2 ns	*Nanoscale* **2023**, *15*, 10820–10825
Spiropyrane-based copolymer	assembly	0.03	~2.5 ns	*J. Mater. Chem. C* **2025**, *13*, 10094–10102
BODIPY derivatives	chiral design	0.0013	7.17 ns	*Angew. Chem. Int. Ed.* **2023**, *62*, e202218023
Quantum dots	chiral design	0.0017	442 μs	*J. Phys. Chem. Lett.* **2024**, *15*, 2049–2056

Lanthanide-based metal complex	chiral design	0.035	124 μs	*J. Am. Chem. Soc.* **2025**, *147*, 1387–1391
Porphyrin-based polymer	assembly	0.0012	1.5 ms	*Green Chem. Eng.* **2025**, *6*, 1–5
Au cyclic trinuclear complexes	chiral design	0.0034	8.6 μs	*Angew. Chem. Int. Ed.* **2023**, *62*, e202310495.
Naphthalimide-based materials	assembly	0.0166	2.2 ms	*J. Mater. Chem. C* **2024**, *12*, 9578-9585
Eu/Cs based metal complex	chiral design	0.00025	NA	*Adv. Sci.* **2024**, *11*, 2305521
BODIPY derivatives	chiral design	0.0059	40 ns	*J. Mater. Chem. C* **2023**, *11*, 2889–2896
Perovskites	chiral design	0.0037	207 ns	*Adv. Mater.* **2025**, *37*, 2413669
Yb based metal complex	chiral design	0.01	NA	*Inorg. Chem.* **2025**, *64*, 5505–5512
Yb,Er based UCNPs	chiral design	0.026	0.68 ms	*Chem. Eng. J.* **2023**, *474*, 145429
Tetrabenzodinaphthopyranthren derivative	chiral design	0.004	132 ms	*Adv. Funct. Mater.* **2024**, *34*, 2308110
Chiral MOFs	chiral design	0.012	150 ms	*Adv. Sci.* **2025**, *12*, 2502784
Organoborane macrocycles	chiral design	0.00067	4.8 ns	*J. Am. Chem. Soc.* **2023**, *145*, 10092–10103

Inorganic phosphors	chiral design	NA	345 µs	*Ceram. Int.* **2024**, *50*, 39823–39829
Diimide dimers	chiral design	0.002	NA	*J. Am. Chem. Soc.* **2024**, *146*, 13499–13508
Metal nanocluster	chiral design	0.0023	12.0 ns	*Chem. Sci.* **2023**, *14*, 7304–7309
UCNPs	assembly	0.12	269 µs	*Sci. China Chem.* **2024**, *67*, 2571–2577
Ln based metal complex	chiral design	0.64	3.91 µs	*Chem. Eur. J.* **2023**, *29*, e202300800
BODIPY derivatives	chiral design	0.047	1.0 ns	*Chem. Mater.* **2024**, *36*, 3745–3753
Binaphthol derivatives	chiral design	0.0049	3.0 ns	*Adv. Optical Mater.* **2024**, *12*, 2302486
Lanthanide-doped nanoparticles	assembly	~0.05	~3.0 ms	*Angew. Chem. Int. Ed.* **2025**, *64*, e202417223
Perovskite	assembly	0.0055	1.58 µs	*Small* **2024**, *20*, 2311639
Quantum dots	assembly	0.20	4.9 ns	*Nano Today* **2024**, *55*, 102197
Er based metal complex	chiral design	0.83	2.7 µs	*J. Mater. Chem. C* **2023**, *11*, 5290–5296
Dicyanobenzene derivatives	assembly	0.0012	1.6 ns	*ACS Appl. Nano Mater.* **2023**, *6*, 5394–5403
Er based metal complex	chiral design	0.66	213 ns	*J. Mater. Chem. C* **2024**,

				12, 4253–4260
UCNPs	assembly	0.6	~200 ms	*Nanoscale* **2025**, *17*, 314–321
Pt complexes	chiral design	0.003	0.7 µs	*Inorg. Chem.* **2024**, *63*, 23642–23650
Helicene derivatives	chiral design	0.0006	20 ns	*Angew. Chem. Int. Ed.* **2024**, *63*, e202412681
Organic–inorganic metal halides	chiral design	0.042	21 µs	*Angew. Chem. Int. Ed.* **2025**, *64*, e202419776
Optical Film	assembly	−1.47	NA	*ACS Nano* **2024**, *18*, 20556–20566
Nile Blue based polymer	assembly	0.045	2.0 ns	*J. Am. Chem. Soc.* **2025**, *147*, 9891–9899
Helicene derivatives	chiral design	0.001	7.0 ns	*J. Am. Chem. Soc.* **2024**, *146*, 10321–10330
Platinum complexes	chiral design	0.0013	1.1 µs	*Sci. China Chem.* **2024**, *67*, 3757–3766
Helical phosphoniums	chiral design	0.0018	~10.0 ns	*Chem. Mater.* **2024**, *36*, 7940–7952
Platinum complexes	chiral design	0.0034	1.9 µs	*Angew. Chem. Int. Ed.* **2023**, *62*, e202302011

NA: not available

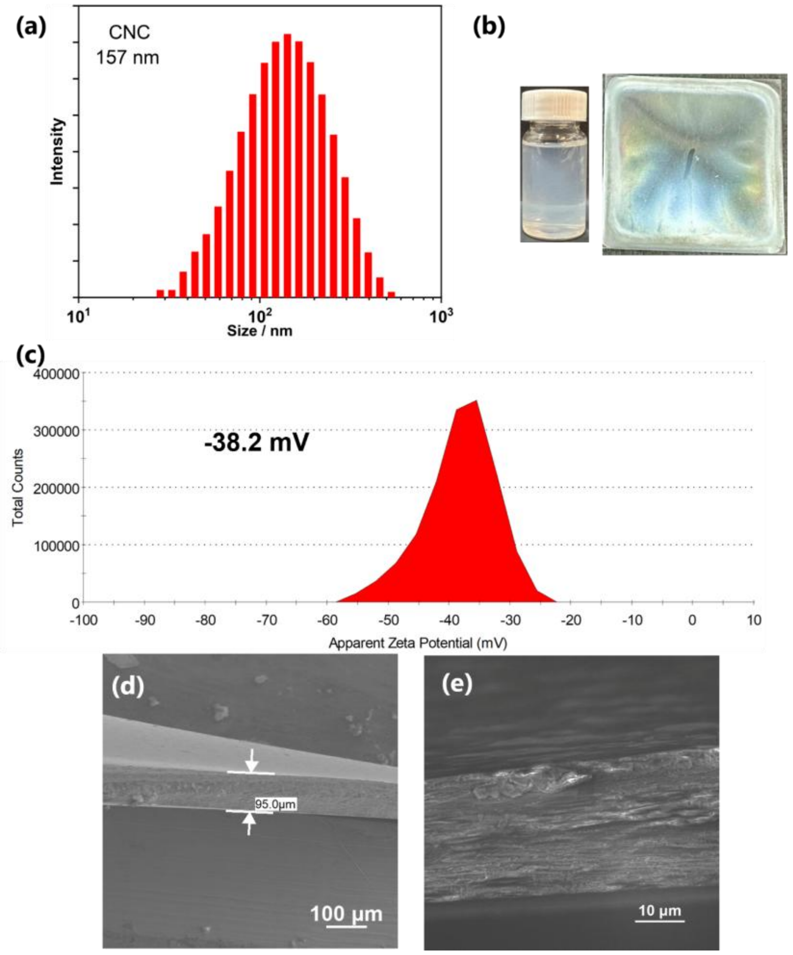

Figure S2. (a) DLS of CNCs solution (1mg/mL). (b) Picture of CNCs solution (3.0 wt%) and CNCs film. (c) Zeta potential of CNCs solution (1 mg/mL). (d,e) SEM images of CNCs films.

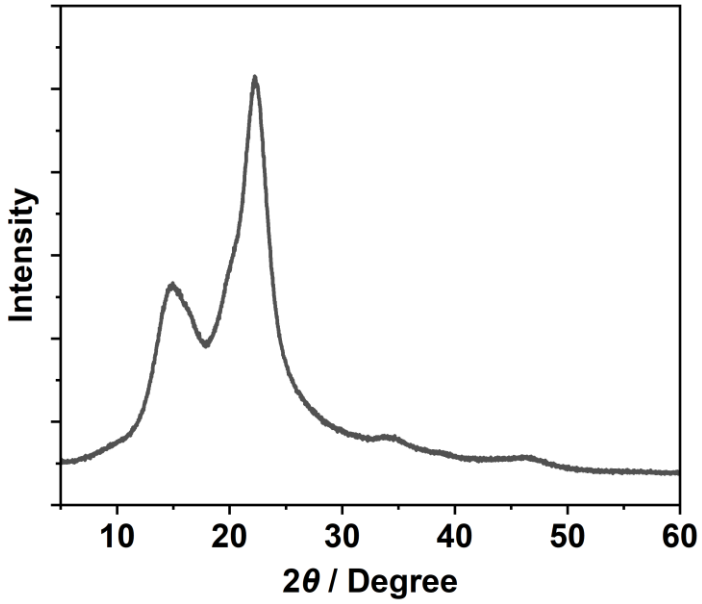

Figure S3. Powder XRD pattern of CNCs film.

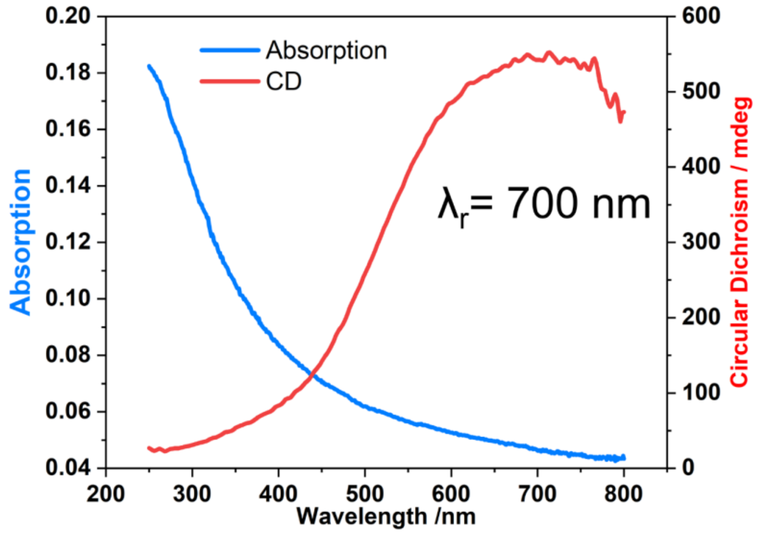

Figure S4. UV–vis absorption and CD spectra of CNCs.

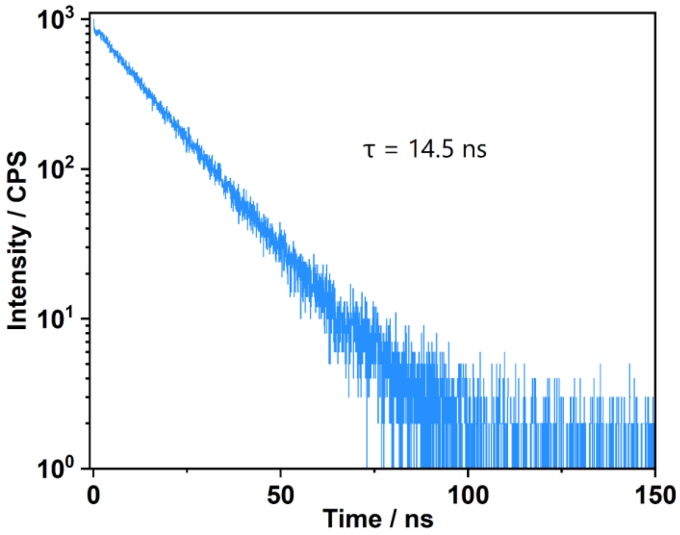

Figure S5. Time-correlated decay curve of TPY/PVA film at 365 nm (m_{TPY} : m_{PVA} = 1 : 100, λ_{ex} = 325 nm).

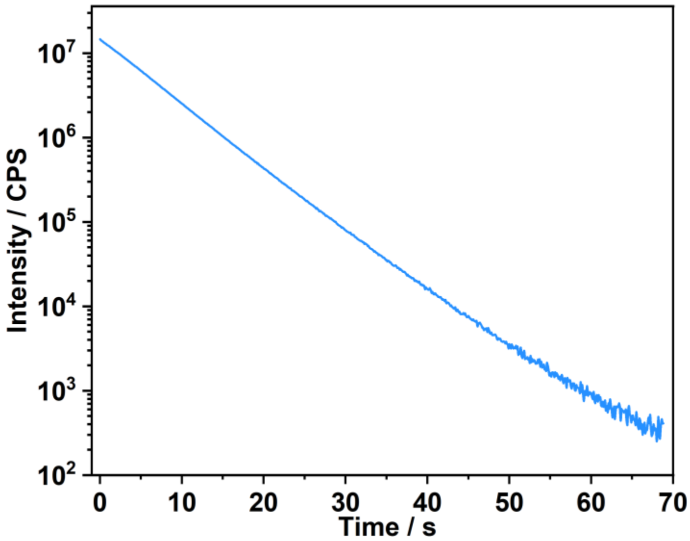

Figure S6. Time-correlated decay curve of TPY/PVA film at 465 nm ($m_{TPY} : m_{PVA} = 1 : 100$, $\lambda_{ex} = 300$ nm).

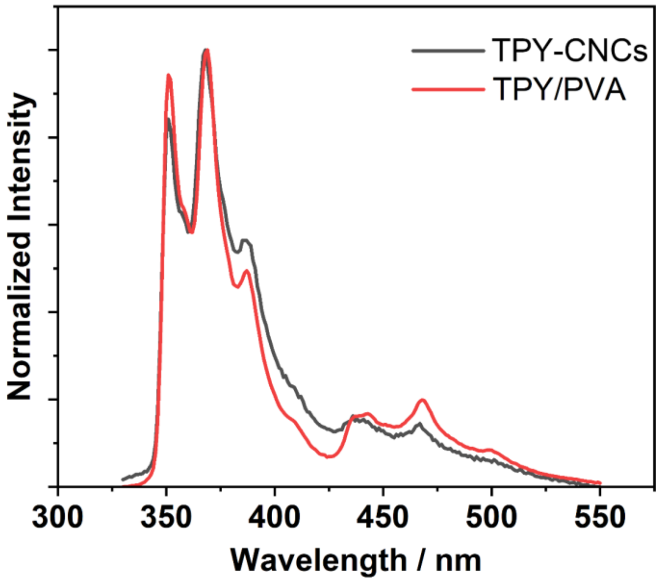

Figure S7. PL spectra of TPY-CNCs film and TPY/PVA film ($\lambda_{ex} = 300$ nm).

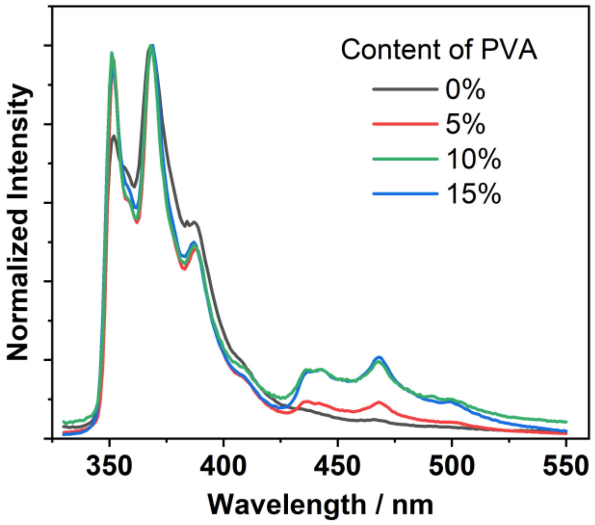

Figure S8. PL spectra of TPY-CNCs films with different PVA contents (λ_{ex} = 300 nm).

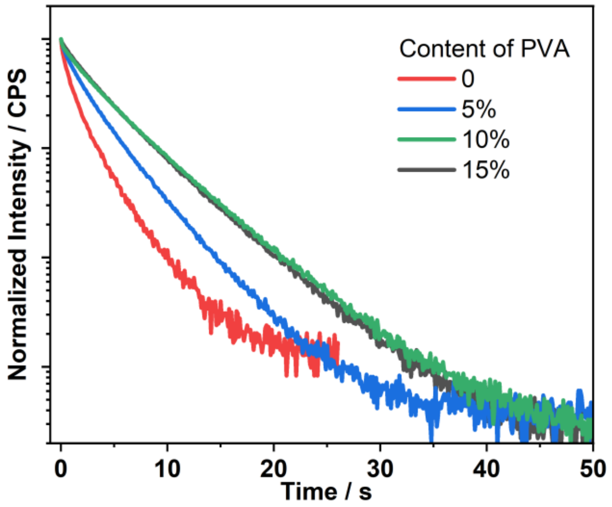

Figure S9. Time-resolved decay spectra of TPY-CNCs films with different PVA contents (λ_{ex} = 300 nm, λ_{em} = 465 nm).

Table S2. PLQY of TPY-CNCs films with different PVA contents (λ_{ex} = 300 nm).

Sample	Φ_{PL}	Φ_{phos}
0	0.24	0.01
5%	0.25	0.03
10%	0.28	0.06
15%	0.29	0.07

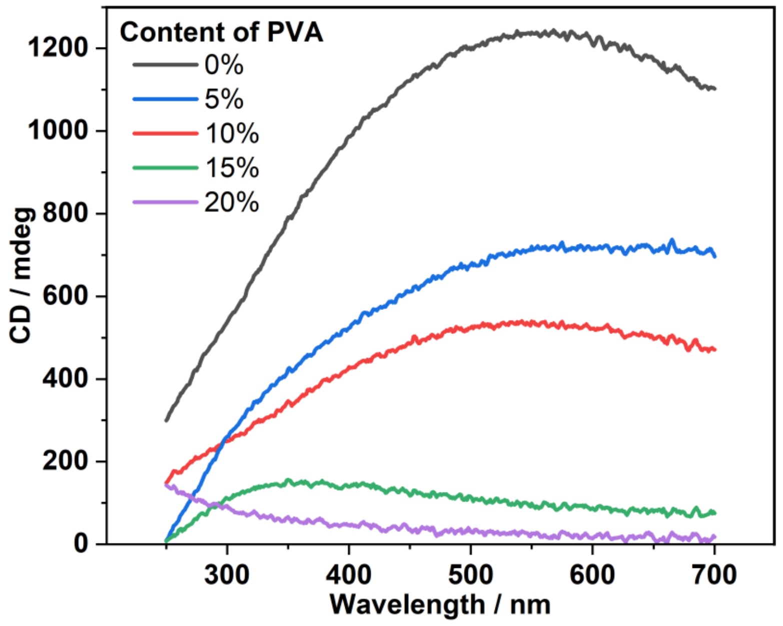

Figure S10. CD spectra of TPY-CNCs films with different PVA contents.

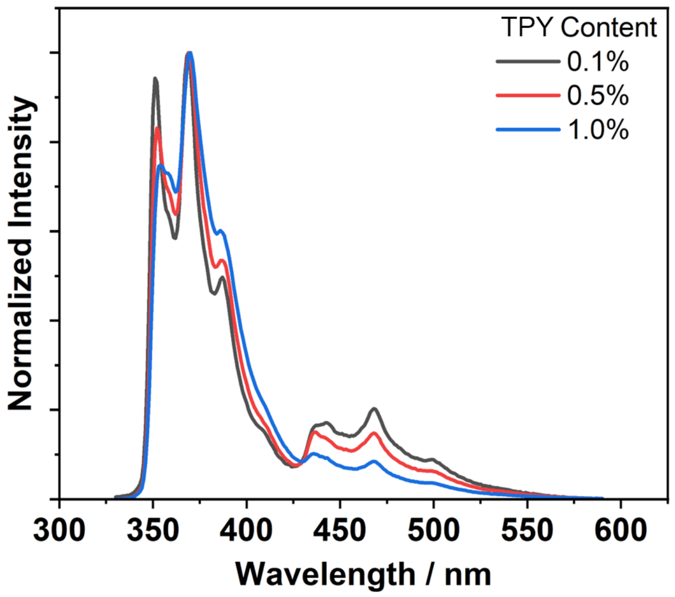

Figure S11. PL spectra of TPY-PCNCs films with different TPY contents (λ_{ex} = 300 nm).

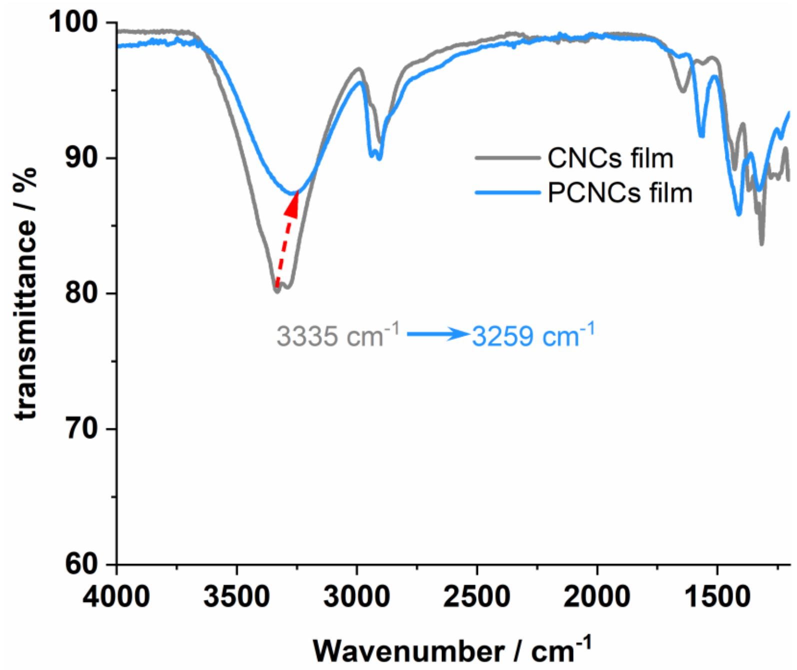

Figure S12. FTIR spectra of CNCs before and after adding PVA.

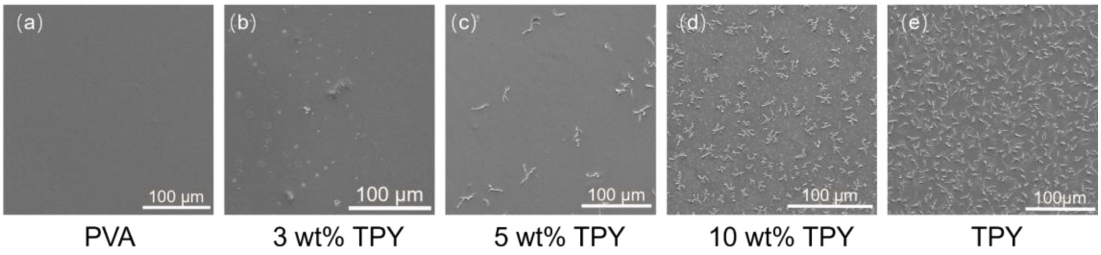

Figure S13. SEM images of TPY/PVA film with various TPY contents.

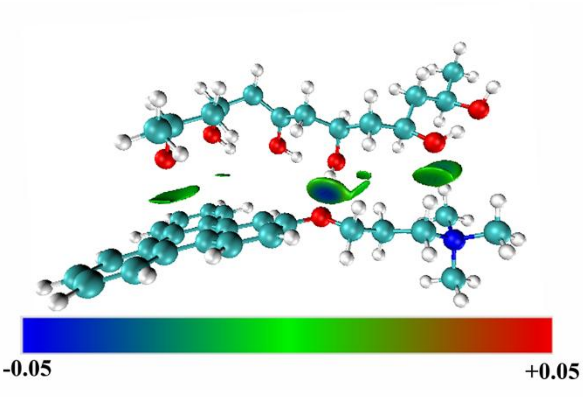

Figure S14. Visualization of the noncovalent interactions in the TPY molecule and PVA polymeric fragment obtained from the Independent Gradient Model based on Hirshfeld partition analysis. The calculation is based on the structure optimized at the B3LYP/6-31G* level. The colored isosurfaces represent different interaction types: blue area (strong attractive interactions such as hydrogen bonds; green area (van der Waals interactions) and red area (strong steric repulsion).

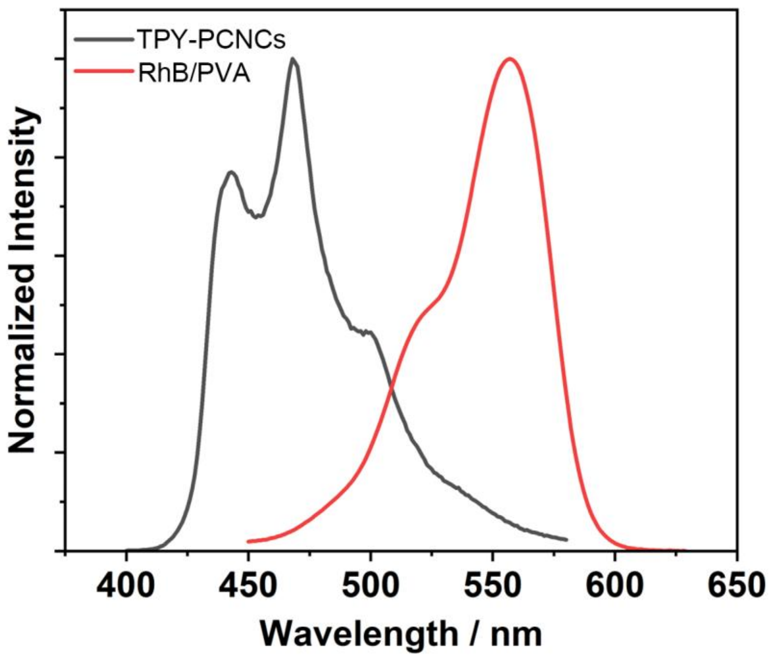

Figure S15. Normalized phosphorescence spectrum of TPY-PCNCs film and normalized absorption spectrum of RhB/PVA film (λ_{ex} = 300 nm, delayed time = 10 ms).

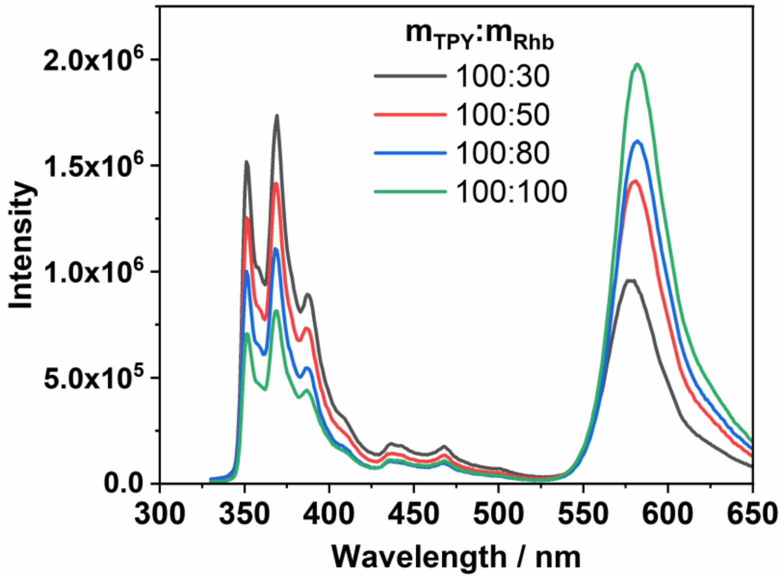

Figure S16. PL spectra of TPY-PCNCs films with different RhB content (λ_{ex} = 300 nm).

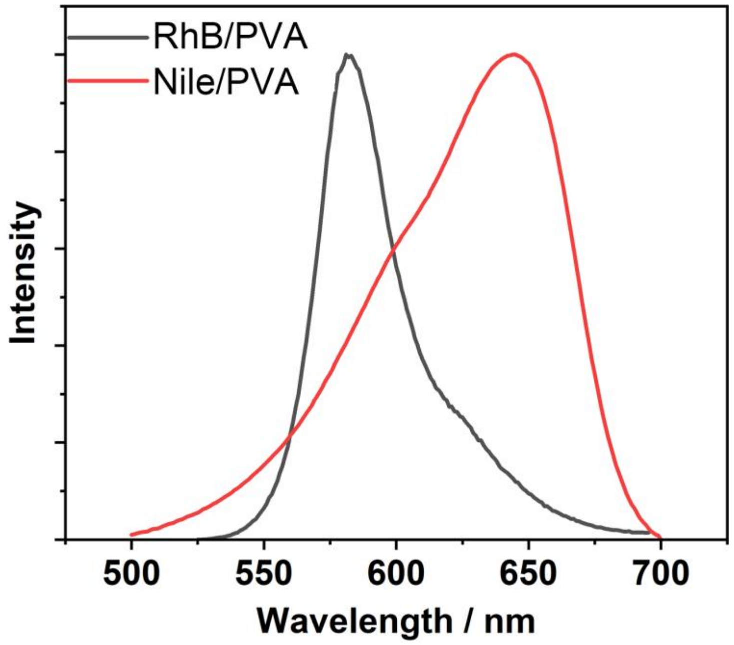

Figure S17. Normalized PL spectrum of RhB/PVA film and normalized absorption spectrum of Nile/PVA film (λ_{ex} = 300 nm).

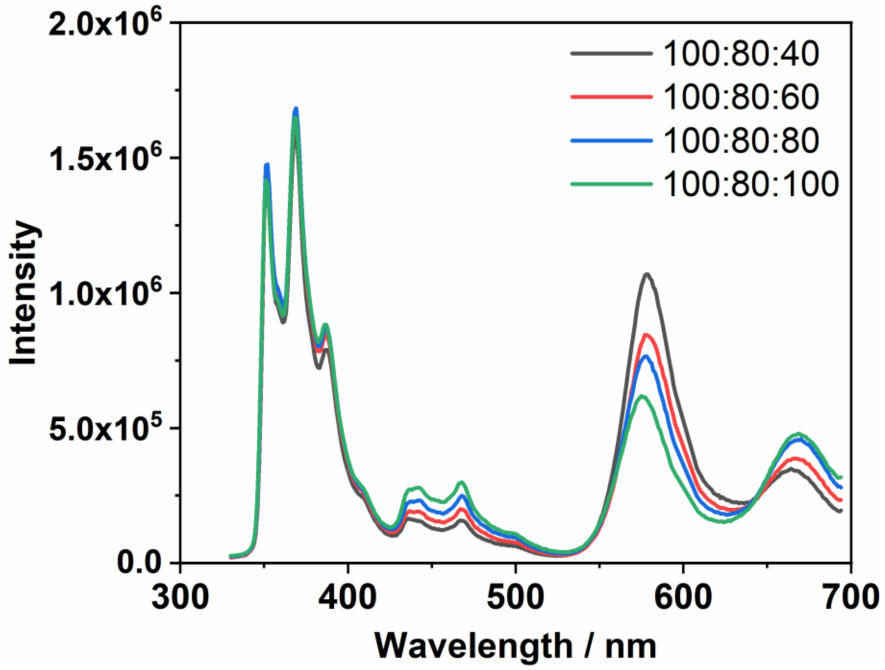

Figure S18. PL spectra of TPY-PCNCs/RhB films with different Nile content (λ_{ex} = 300 nm).

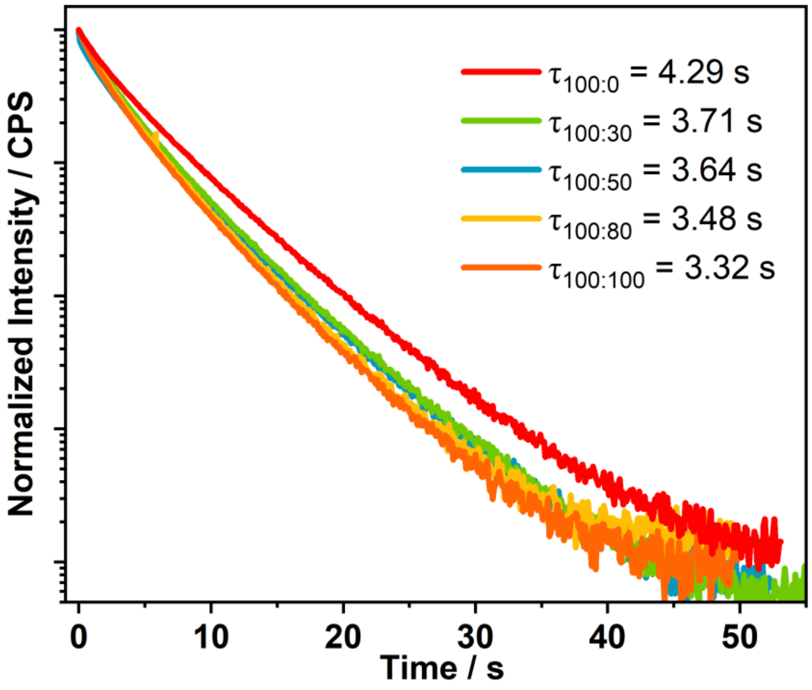

Figure S19. Time-resolved decay spectra of TPY-PCNCs films with different RhB contents (λ_{ex} = 300 nm, λ_{em} = 465 nm).

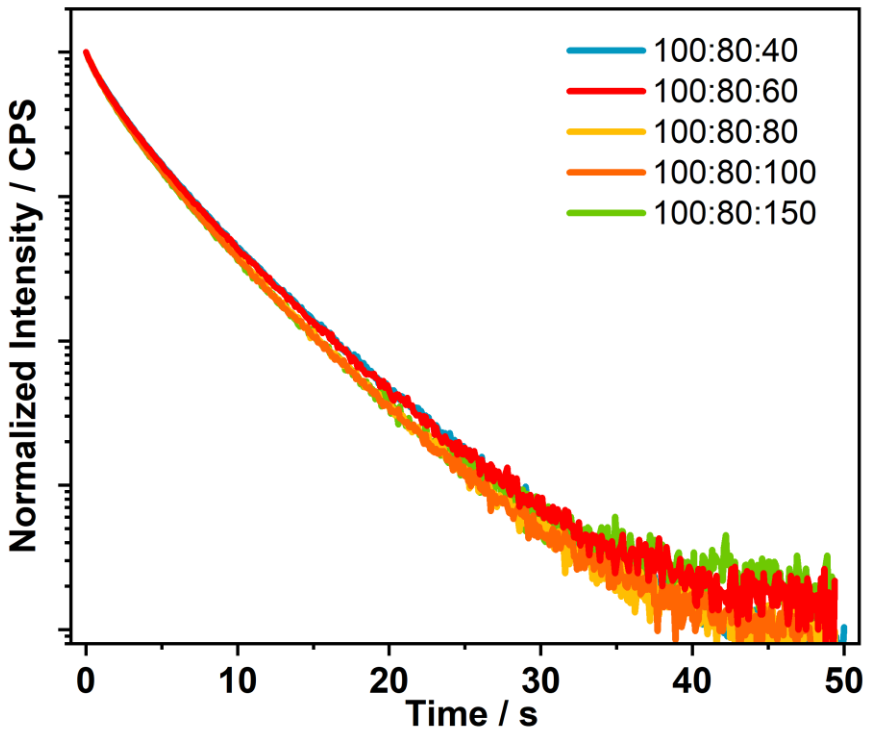

Figure S20. Time-resolved decay spectra of TPY-PCNCs/RhB films with different Nile contents (λ_{ex} = 300 nm, λ_{em} = 465 nm).

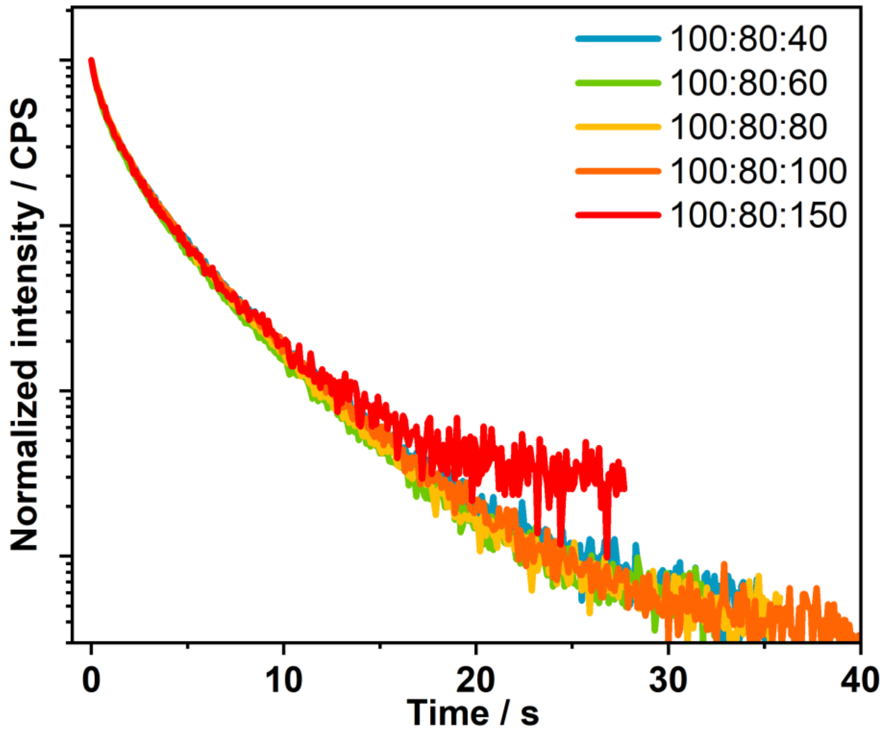

Figure S21. Time-resolved decay spectra of TPY-PCNCs/RhB films with different Nile contents (λ_{ex} = 300 nm, λ_{em} = 580 nm).

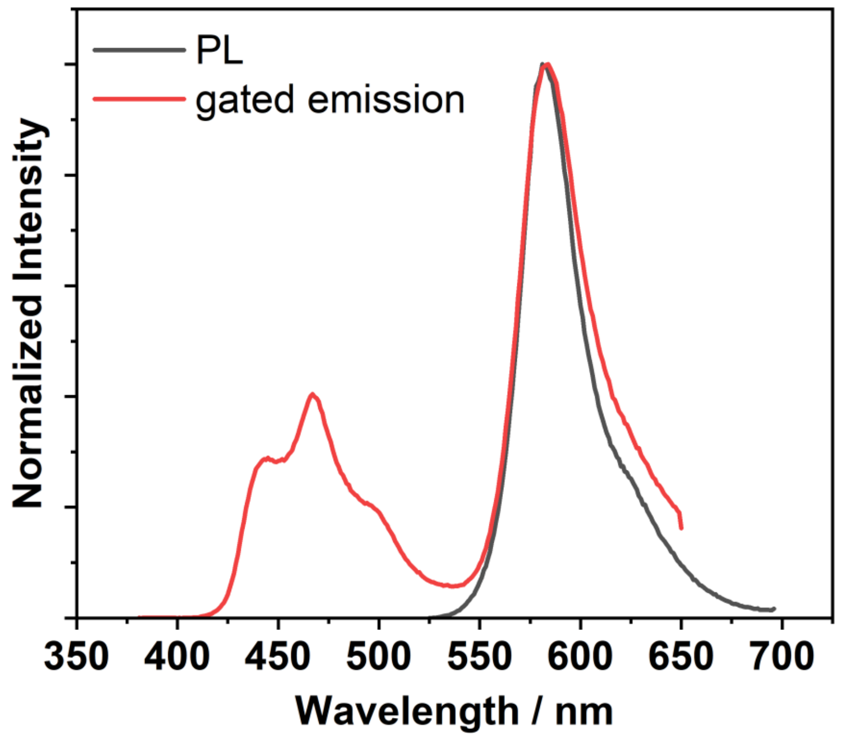

Figure S22. Normalized gated emission spectrum of TPY-PCNCs/RhB film and normalized PL spectrum of RhB/PVA film (λ_{ex} = 300 nm).

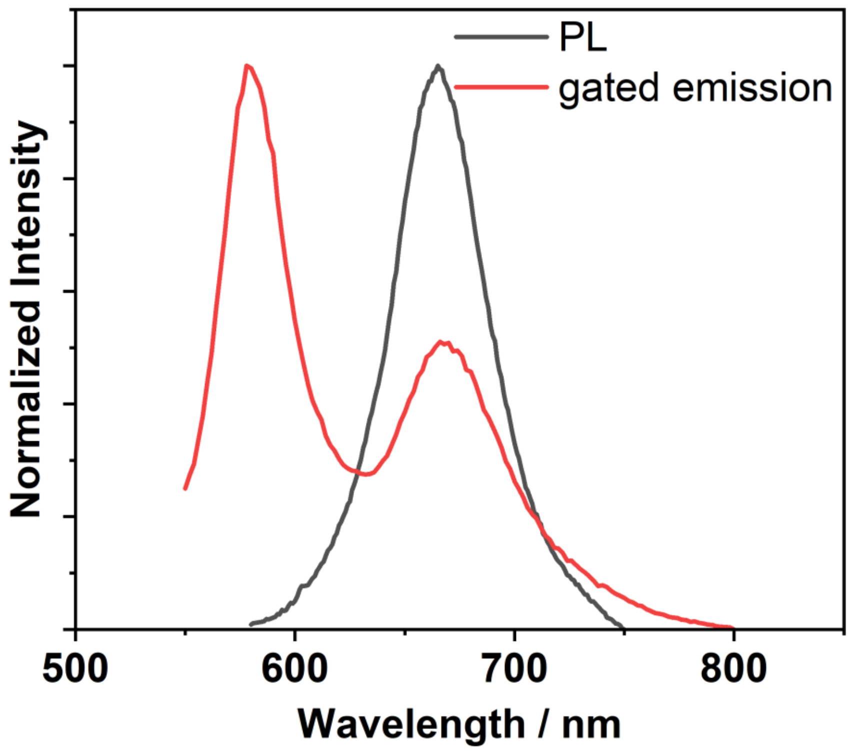

Figure S23. Normalized gated emission spectrum of TPY-PCNCs/RhB@Nile film (λ_{ex} = 300 nm) and normalized PL spectrum of Nile/PVA film (λ_{ex} = 500 nm).

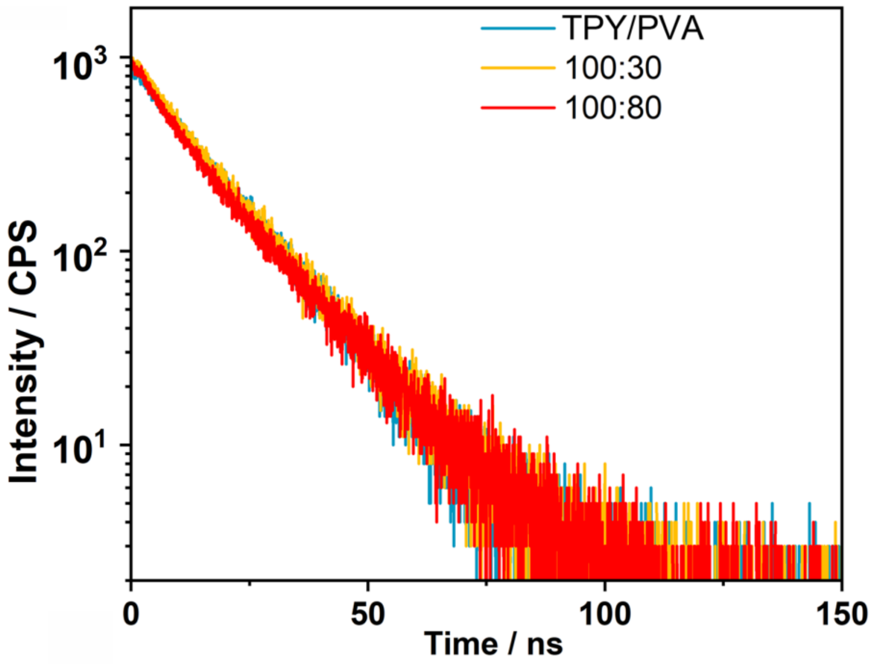

Figure S24. Time-resolved decay spectra of TPY-PCNCs films with different RhB contents and TPY/PVA film (λ_{ex} = 300 nm, λ_{em} = 390 nm).

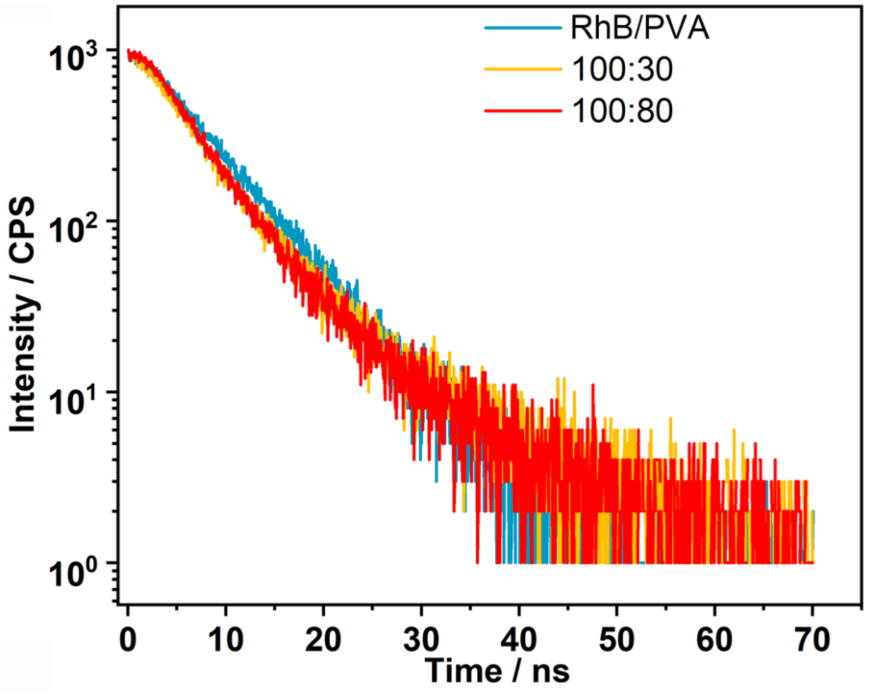

Figure S25. Time-resolved decay spectra of TPY-PCNCs films with different RhB contents and RhB/PVA film (λ_{ex} = 300 nm, λ_{em} = 580 nm).

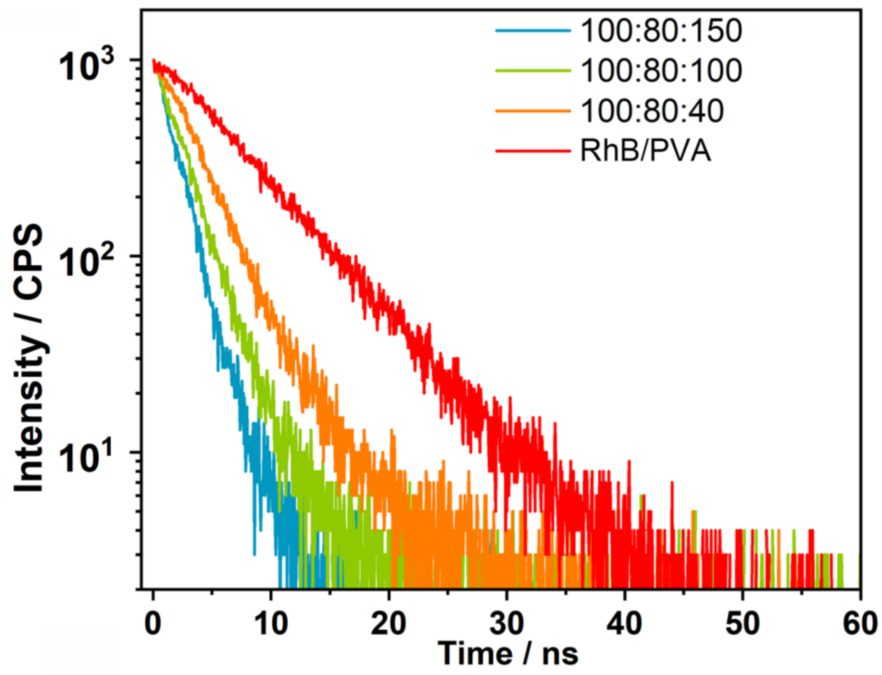

Figure S26. Time-resolved decay spectra of TPY-PCNCs/RhB films with different Nile contents and RhB/PVA film (λ_{ex} = 300 nm, λ_{em} = 580 nm).

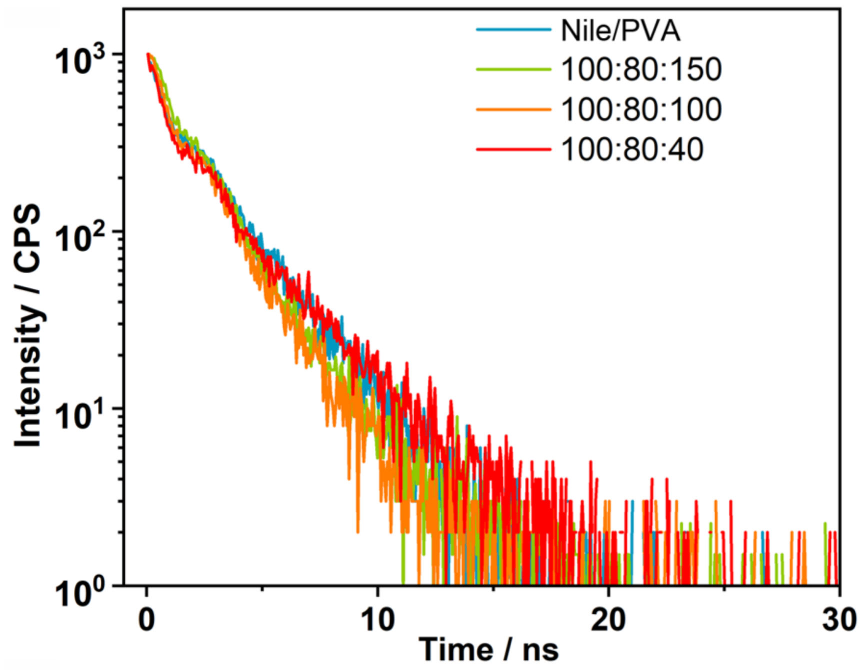

Figure S27. Time-resolved decay spectra of TPY-PCNCs/RhB films with different Nile contents and Nile/PVA film (λ_{ex} = 300 nm, λ_{em} = 580 nm).

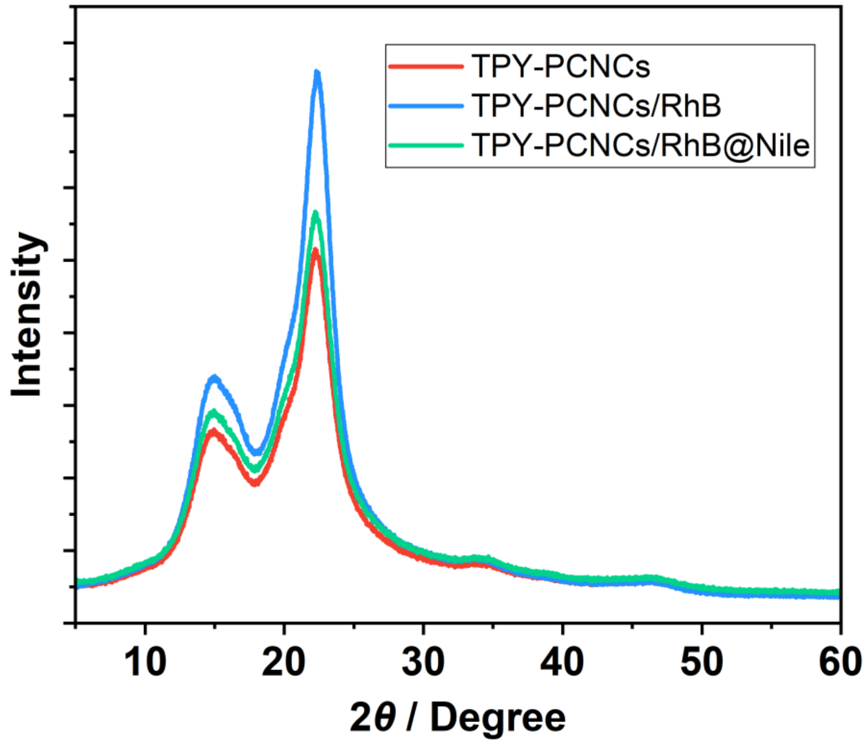

Figure S28. Powder XRD patterns of TPY-PCNCs, TPY-PCNCs/RhB and TPY-PCNCs/RhB@Nile films.

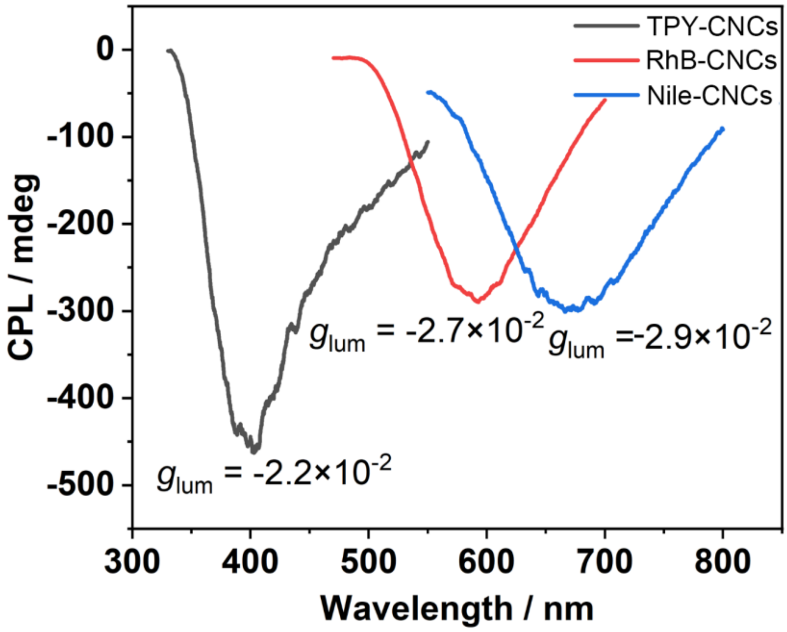

Figure S29. CPL spectra of TPY-CNCs, RhB-CNCs and Nile-CNCs films (λ_{ex} = 300 nm).

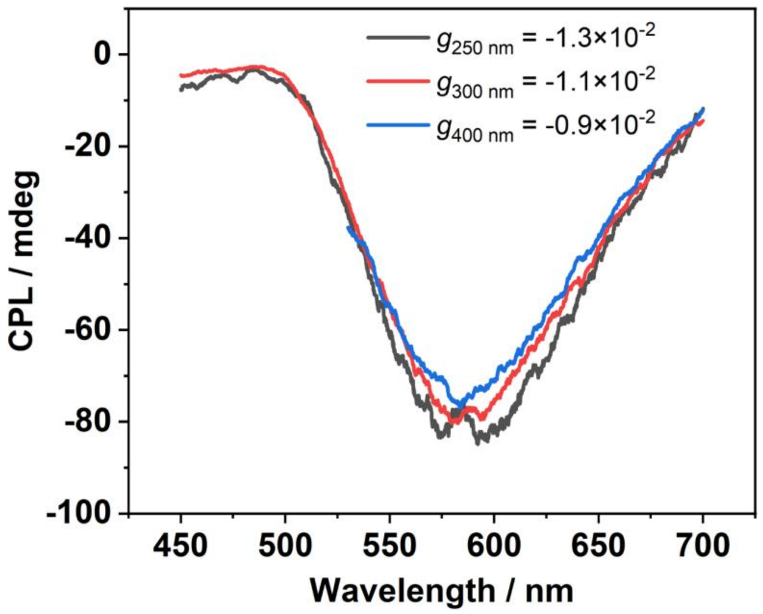

Figure S30. CPL spectra of TPY-PCNCs/RhB films with different excitation wavelength.

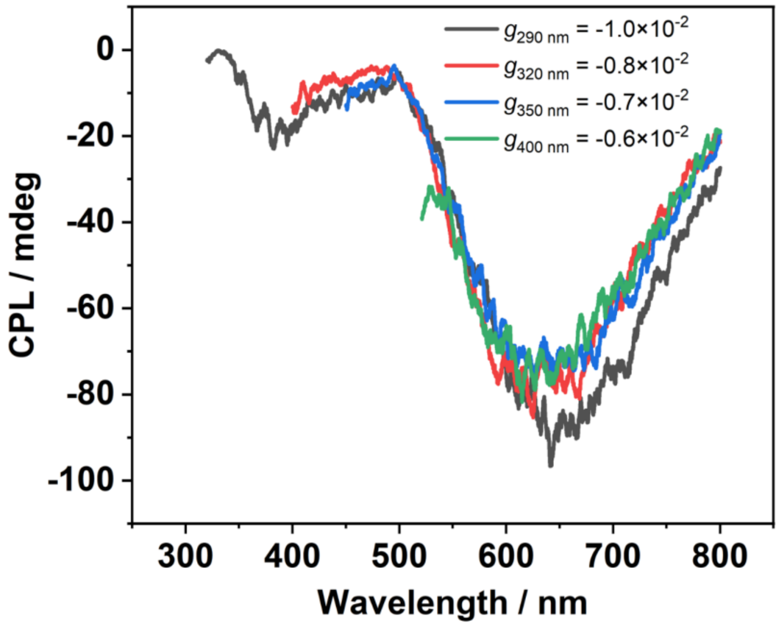

Figure S31. CPL spectra of TPY-PCNCs/RhB@Nile films with different excitation wavelength.

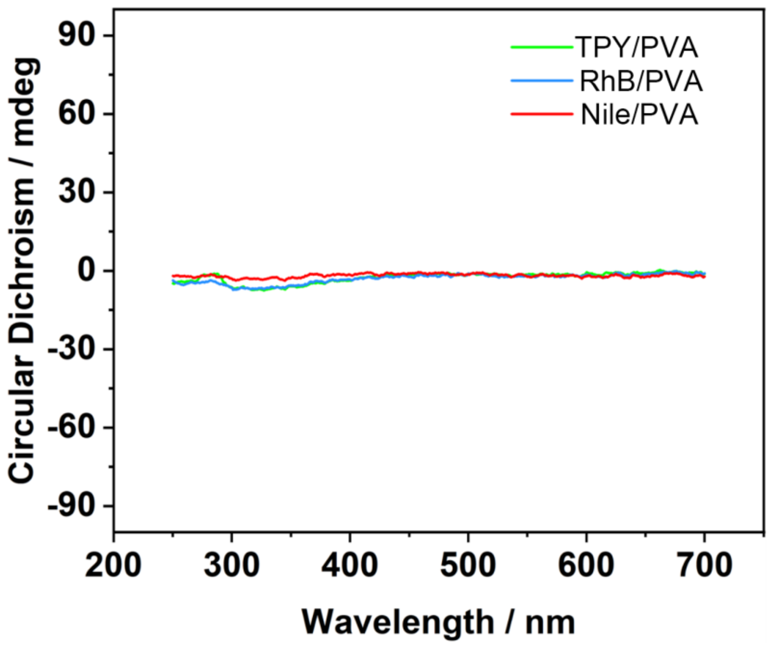

Figure S32. CD spectra of TPY/PVA, RhB/PVA, and Nile/PVA films.

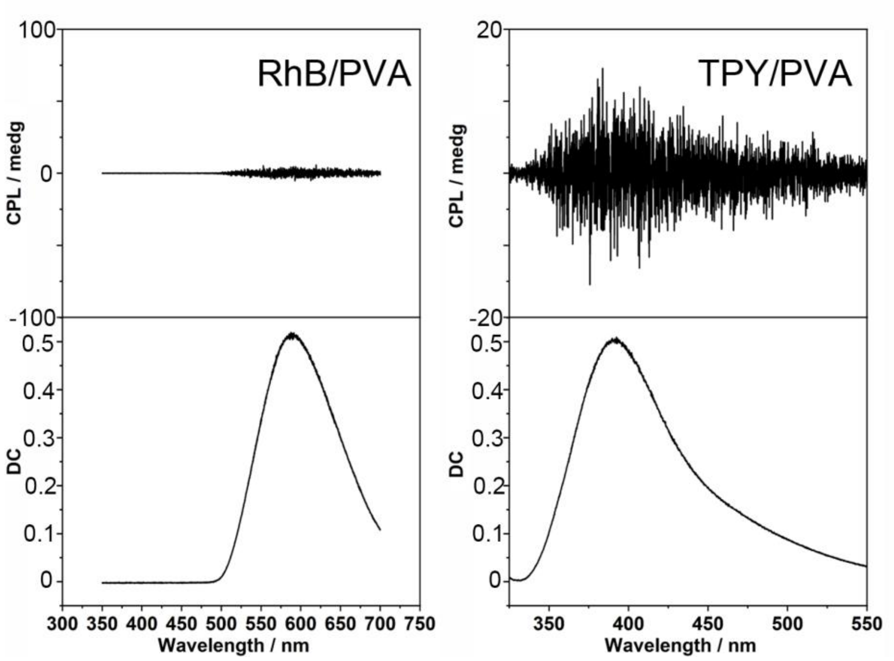

Figure S33. CPL spectra of RhB/PVA and TPY/PVA films (λ_{ex} = 300 nm).

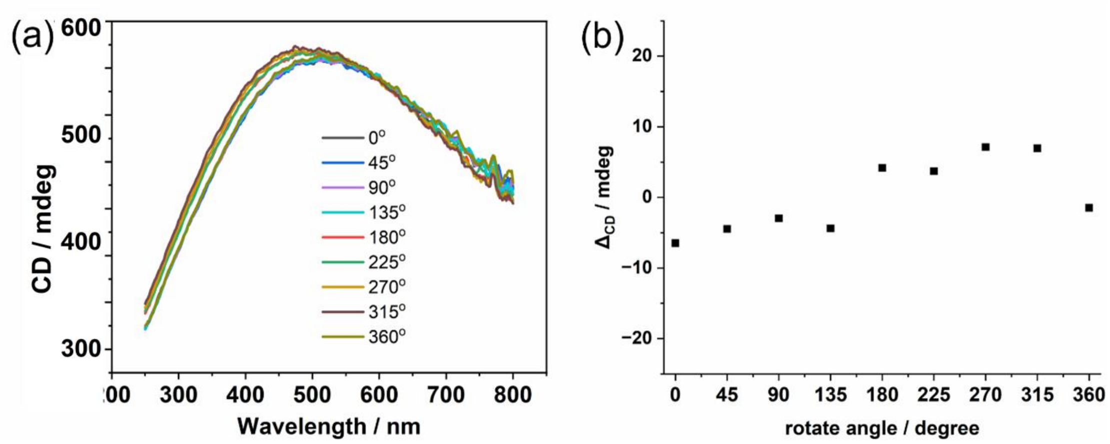

Figure S34. (a) Angle-dependent CD spectra of PCNCs when rotating the sample about the optical axis in steps of 45°; (b) Difference between the CD value and the average value for different angles at 500 nm.

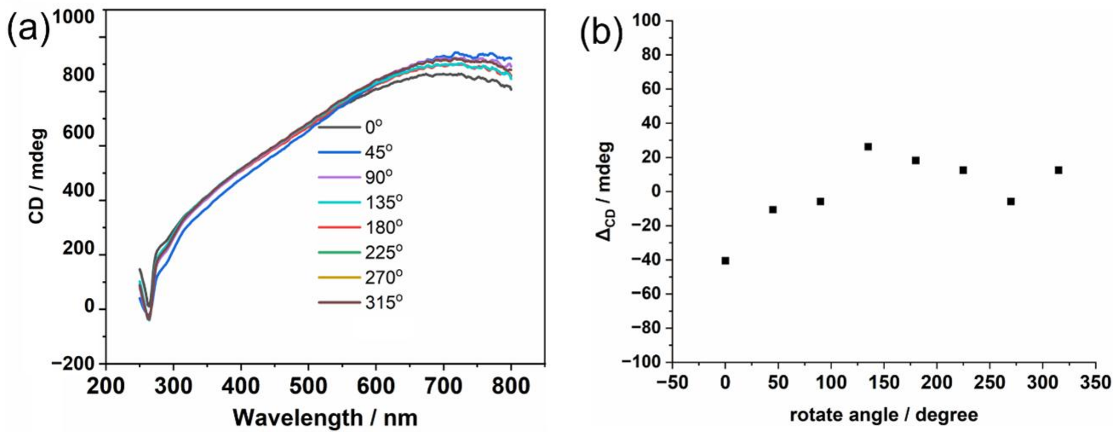

Figure S35. (a) Angle-dependent CD spectra of TPY-PCNCs when rotating the sample about the optical axis in steps of 45°; (b) Difference between the CD value and the average value for different angles at 700 nm.

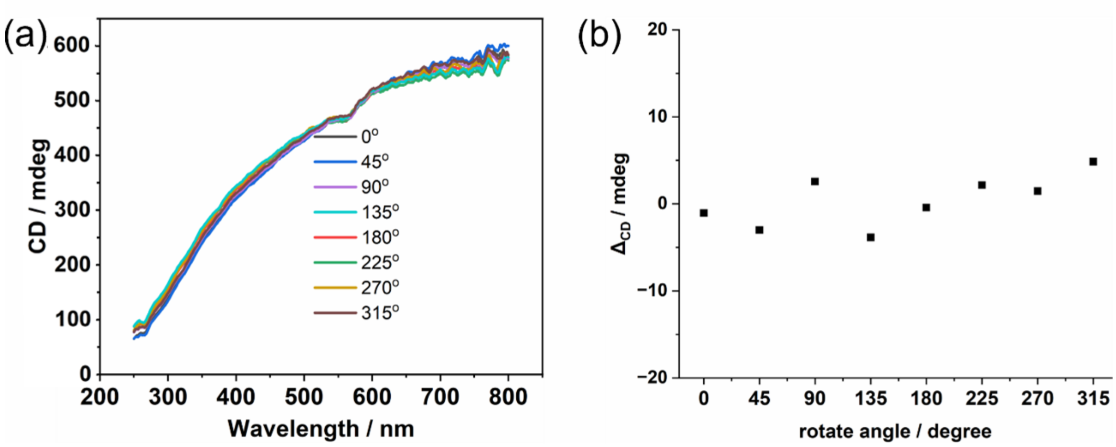

Figure S36. (a) Angle-dependent CD spectra of TPY-PCNCs/RhB when rotating the sample about the optical axis in steps of 45°; (b) Difference between the CD value and the average value for different angles at 700 nm.

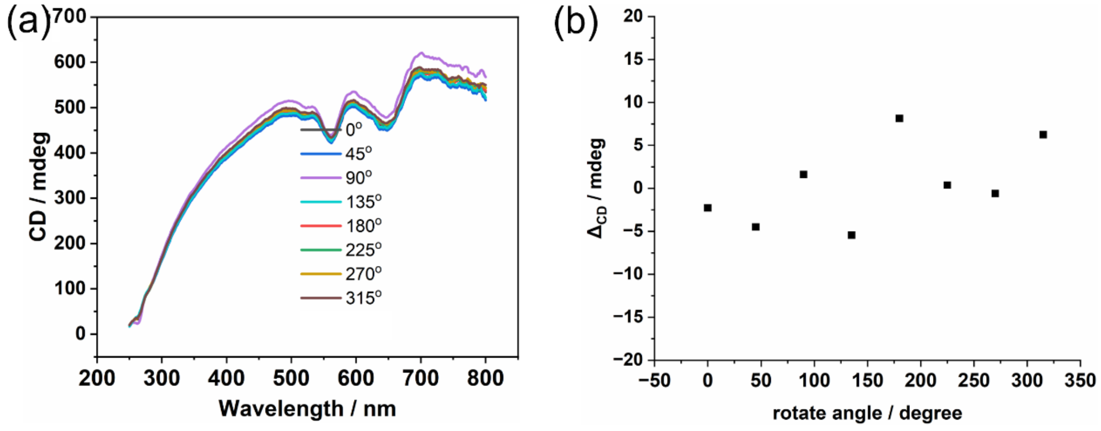

Figure S37. (a) Angle-dependent CD spectra of TPY-PCNCs/RhB@Nile when rotating the sample about the optical axis in steps of 45°; (b) Difference between the CD value and the average value for different angles at 550 nm.

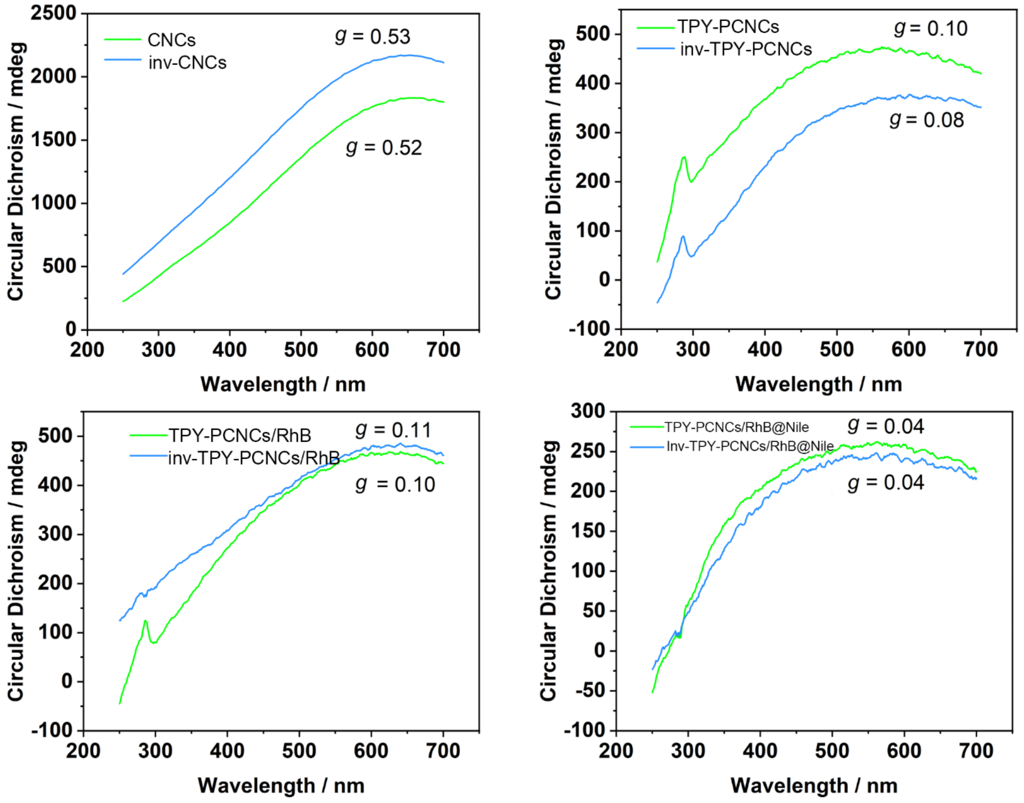

Figure S38. CD spectra of CNCs, TPY-PCNCs, TPY-PCNCs/RhB and TPY-PCNCs/RhB@Nile films before and after being inversed (inv means inversed films).

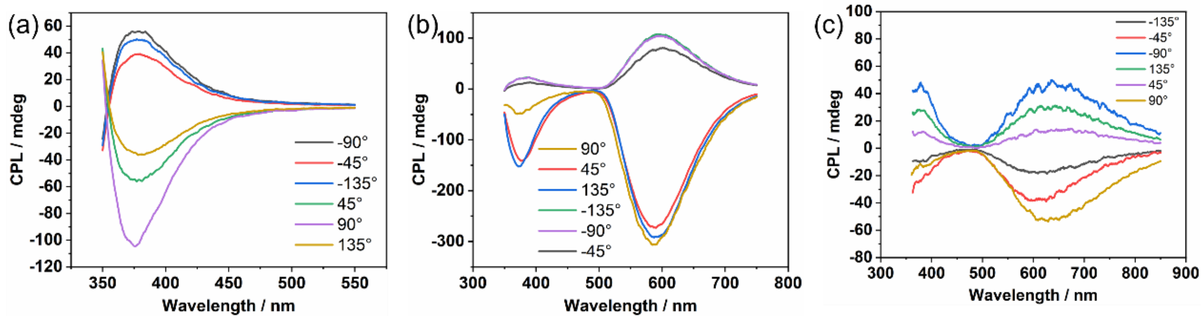

Figure S39. CPL spectra with different incident light angles of (a) TPY-PCNCs films, (b) TPY-PCNCs/RhB films, and (c) TPY-PCNCs/RhB@Nile films (λ_{ex}= 280 nm).

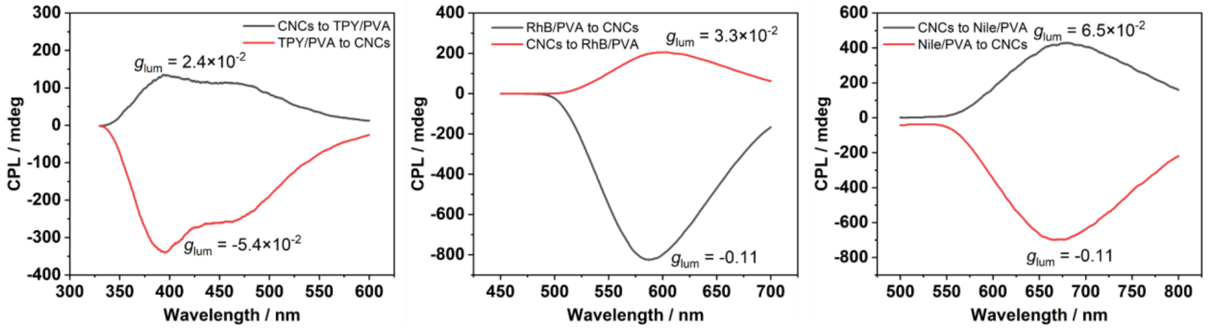

Figure S40. CPL spectra of CNCs along with TPY/PVA, CNCs along with RhB/PVA and CNCs along with Nile/PVA ("CNCs to TPY/PVA" means CNCs films are at lamp side and TPY/PVA films are at detector side, λ_{ex} = 300 nm or 400 nm).

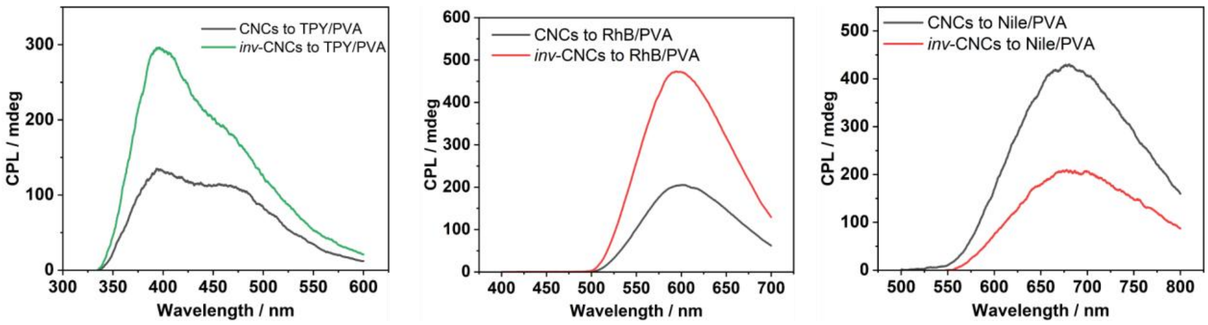

Figure S41. CPL spectra of CNCs along with TPY/PVA, CNCs along with RhB/PVA and CNCs along with Nile/PVA before and after being inversed (inv means inversed films; "CNCs to TPY/PVA" means CNCs films are at lamp side and TPY/PVA films are at detector side, λ_{ex} = 300 nm or 400 nm).

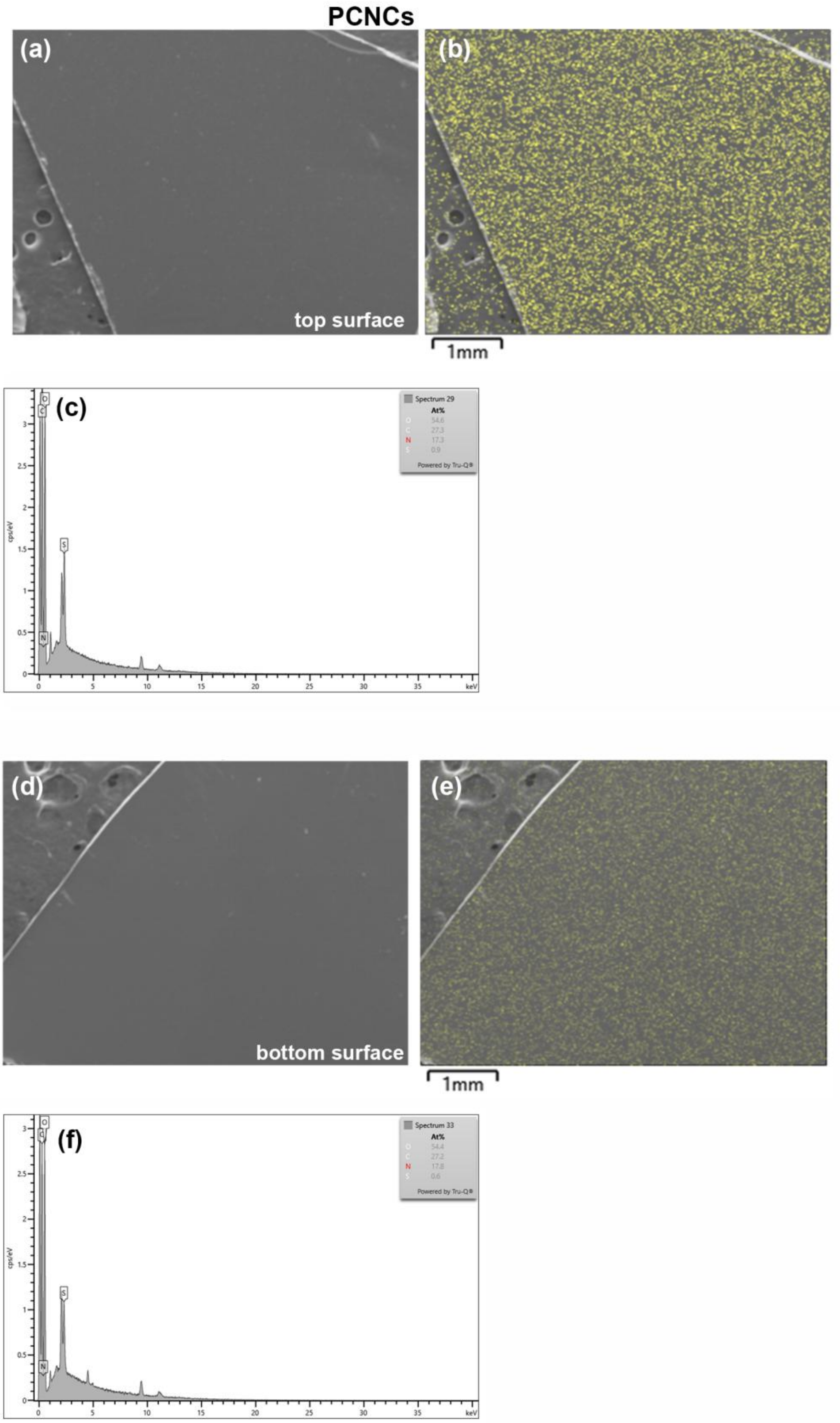

Figure S42. SEM images of (a) PCNCs top surface and (d) PCNCs bottom surface. Corresponding EDS mapping images of (b) PCNCs top surface and (e) PCNCs bottom surface (elemental map of S). EDS spectra of (c) PCNCs top surface and (f) PCNCs bottom surface.

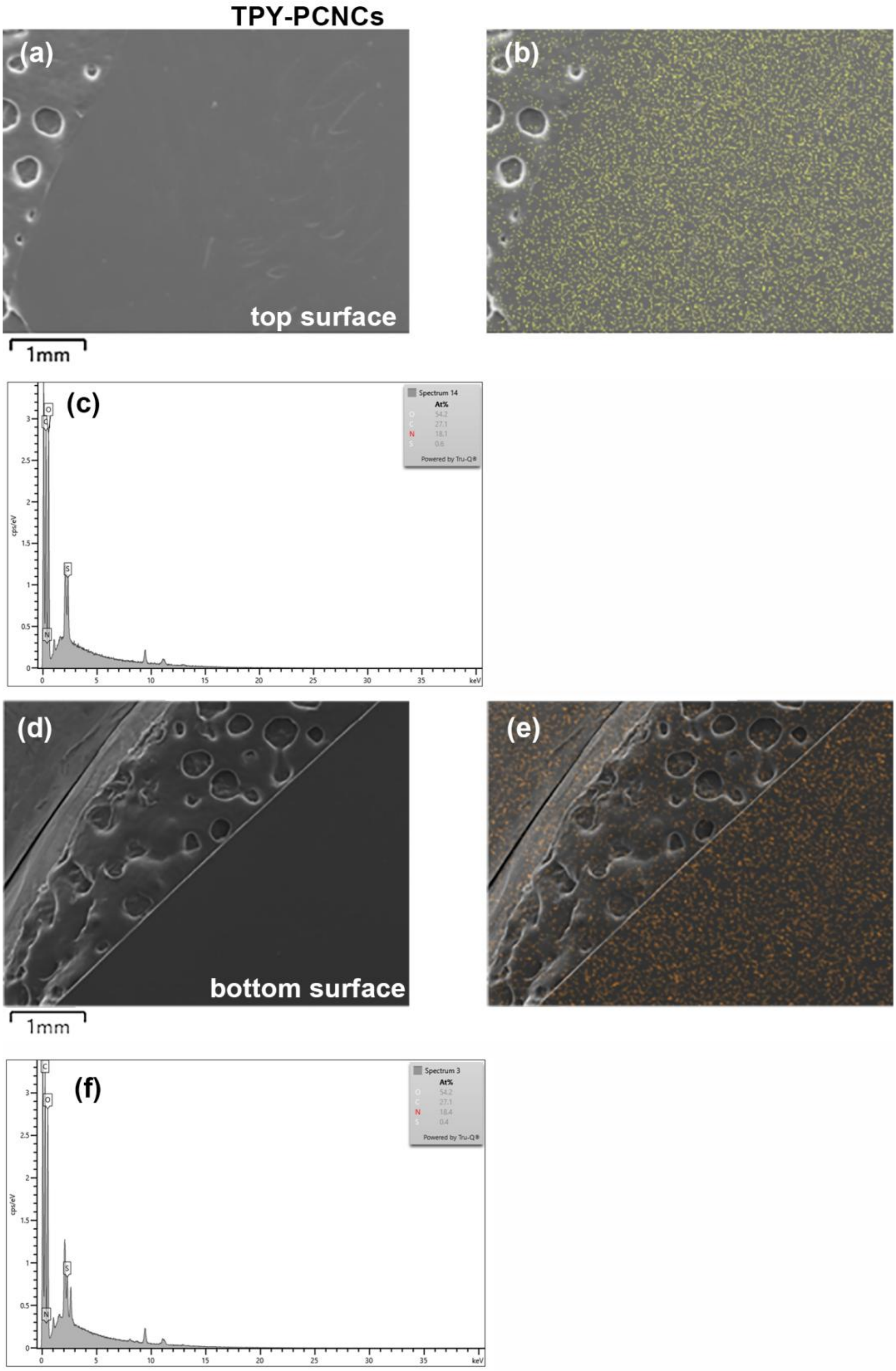

Figure S43. SEM images of (a) TPY-PCNCs top surface and (d) TPY-PCNCs bottom surface. Corresponding EDS mapping images of (b) TPY-PCNCs top surface and (e) TPY-PCNCs bottom surface (elemental map of S). EDS spectra of (c) TPY-PCNCs top surface and (f) TPY-PCNCs bottom surface.

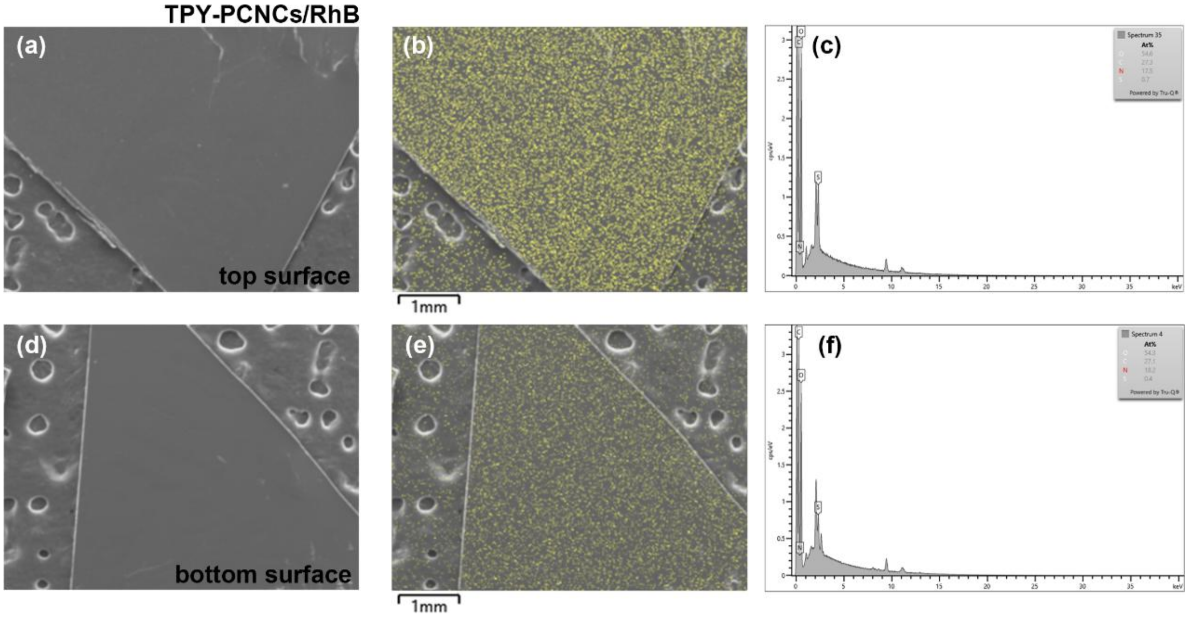

Figure S44. SEM images of (a) TPY-PCNCs/RhB top surface and (d) TPY-PCNCs/RhB bottom surface. Corresponding EDS mapping images of (b) TPY-PCNCs/RhB top surface and (e) TPY-PCNCs/RhB bottom surface (elemental map of S). EDS spectra of (c) TPY-PCNCs/RhB top surface and (f) TPY-PCNCs/RhB bottom surface.

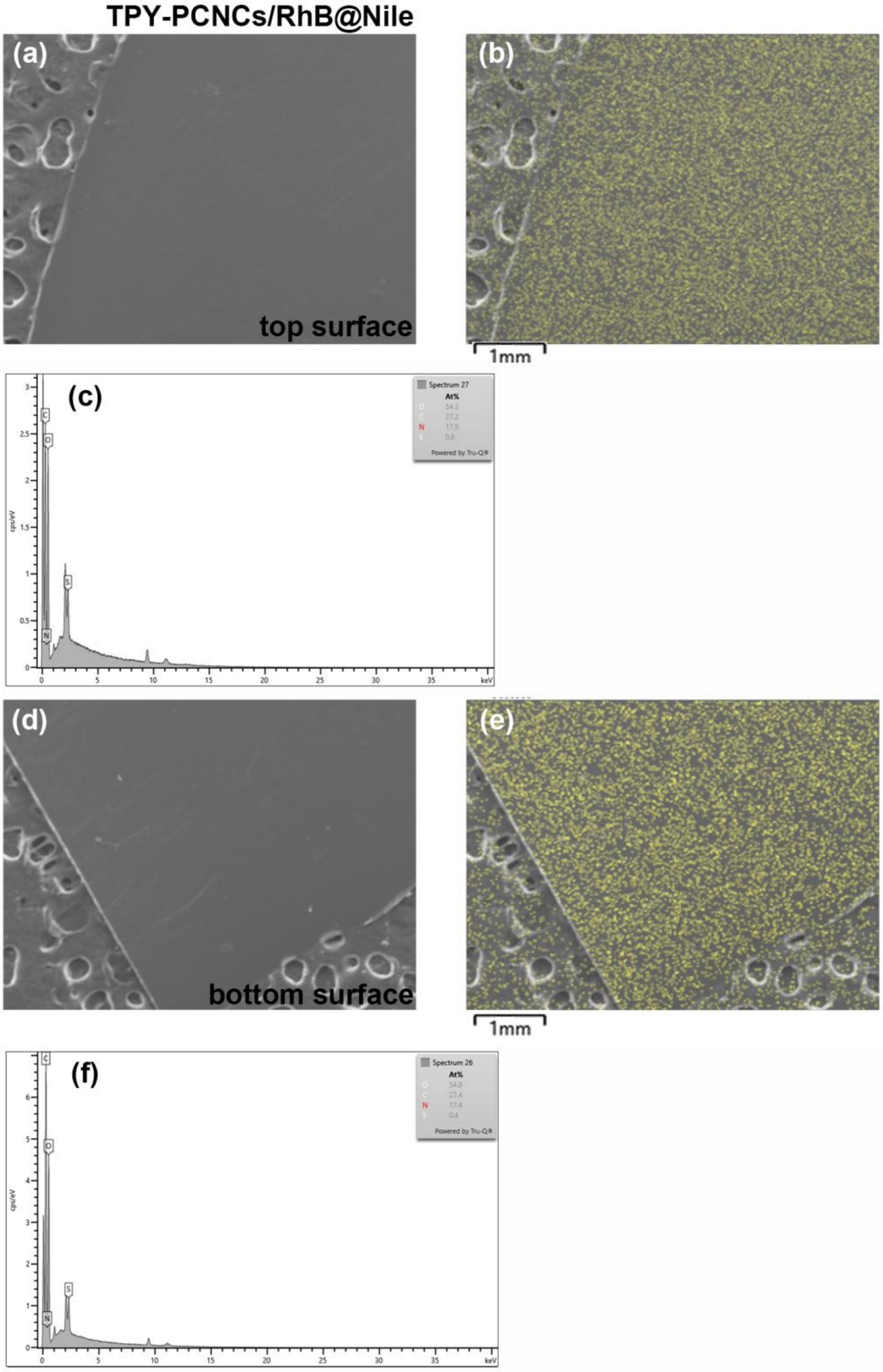

Figure S45. SEM images of (a) TPY-PCNCs/RhB@Nile top surface and (d) TPY-PCNCs/RhB@Nile bottom surface. Corresponding EDS mapping images of (b) TPY-PCNCs/RhB@Nile top surface and (e) TPY-PCNCs/RhB @Nile bottom surface (elemental map of S). EDS spectra of (c) TPY-PCNCs/RhB@Nile top surface and (f) TPY-PCNCs/RhB@Nile bottom surface.

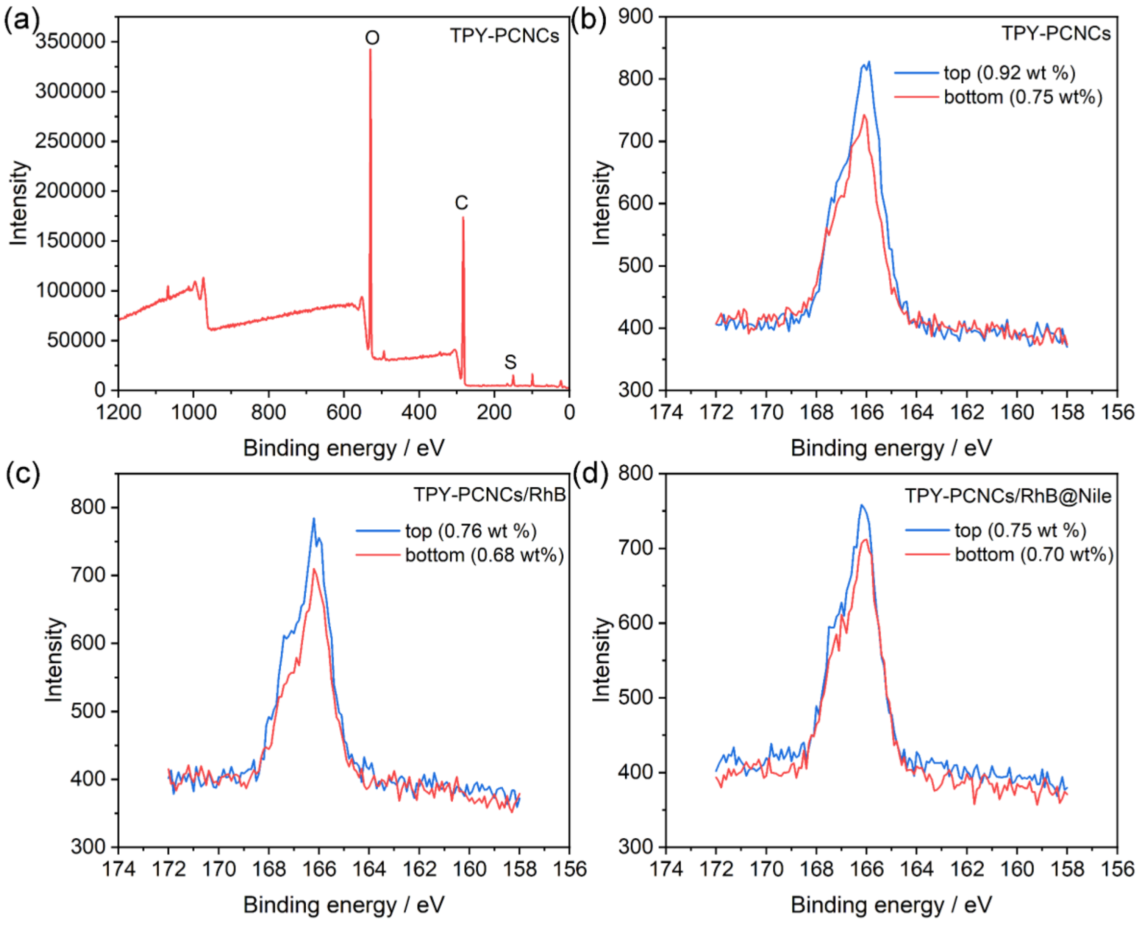

Figure S46. (a) XPS spectra of TPY-PCNCs, and enlarged XPS spectra of (b) TPY-PCNCs, (c) TPY-PCNCs/RhB and (d) TPY-PCNCs/RhB@Nile film.

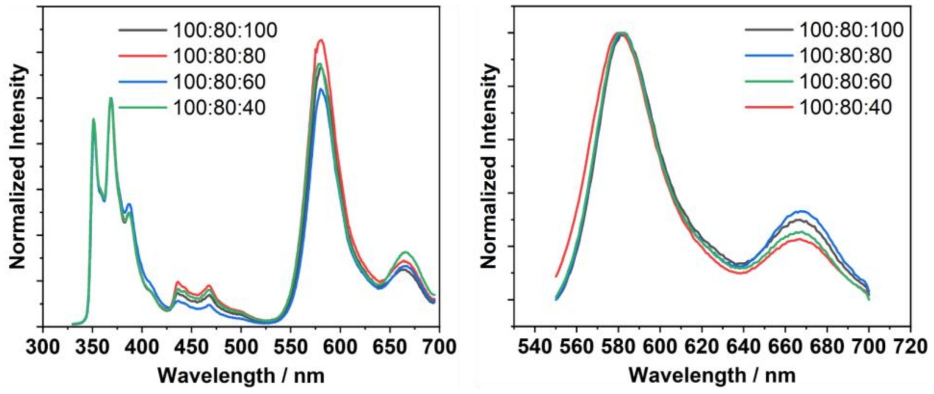

Figure S47. Left: PL spectra of TPY-PCNCs/RhB films with different Cy5 content (λ_{ex} = 300 nm). Right: gated emission spectra of TPY-PCNCs/RhB films with different Cy5 content (λ_{ex} = 300 nm).

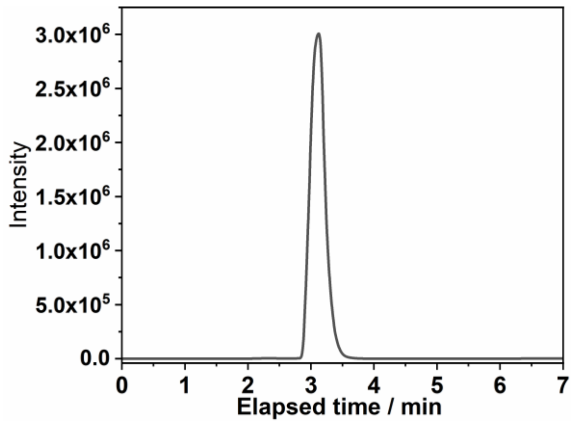

Figure S48. HPLC spectrum of TPY (the eluent being 90/10 H_2O: ACN).

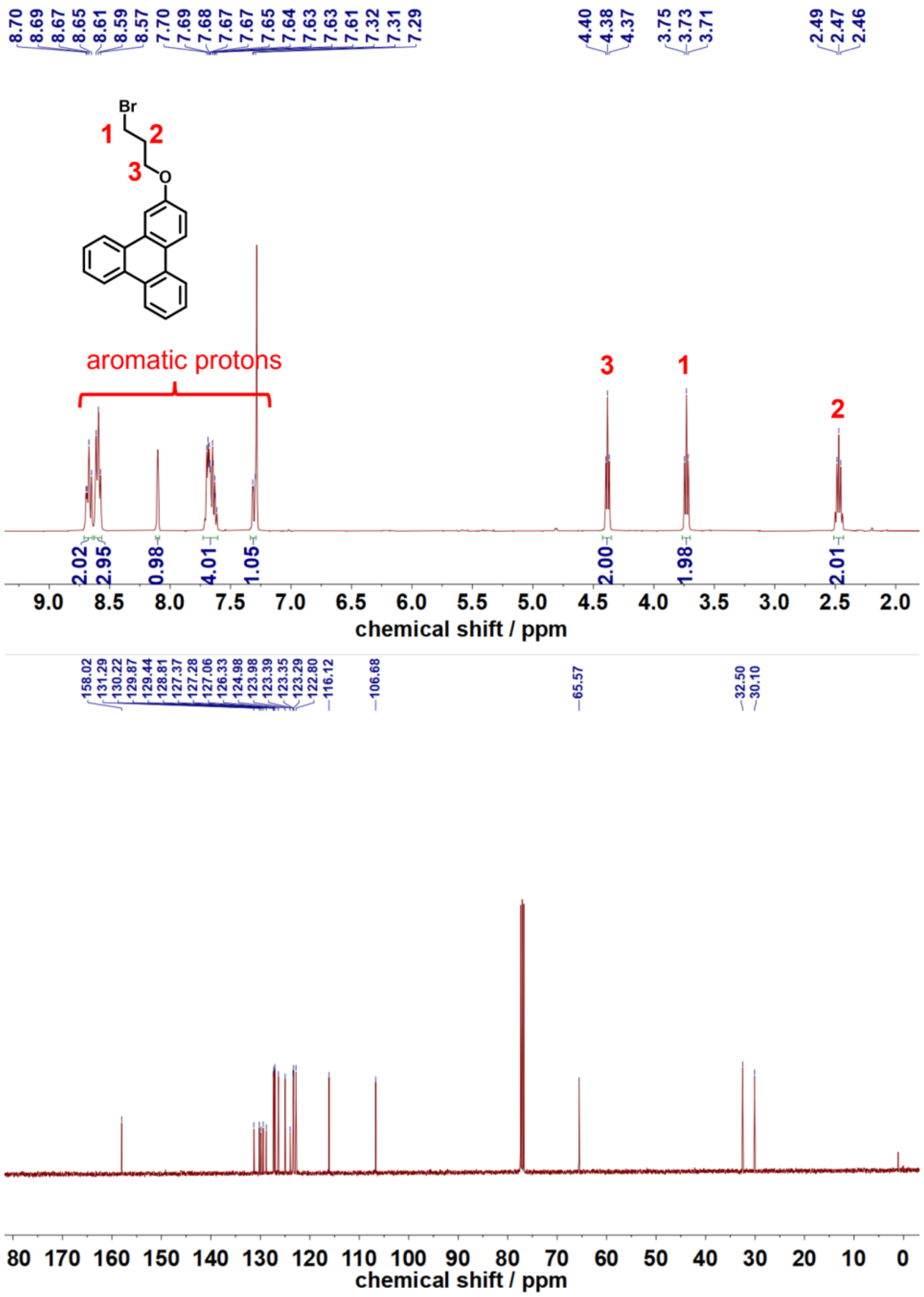

Figure S49. ^{1}H NMR spectrum (400 MHz, $CDCl_3$, 298 K) and ^{13}C NMR spectrum (100 MHz, $CDCl_3$, 298 K) of compound **2**.

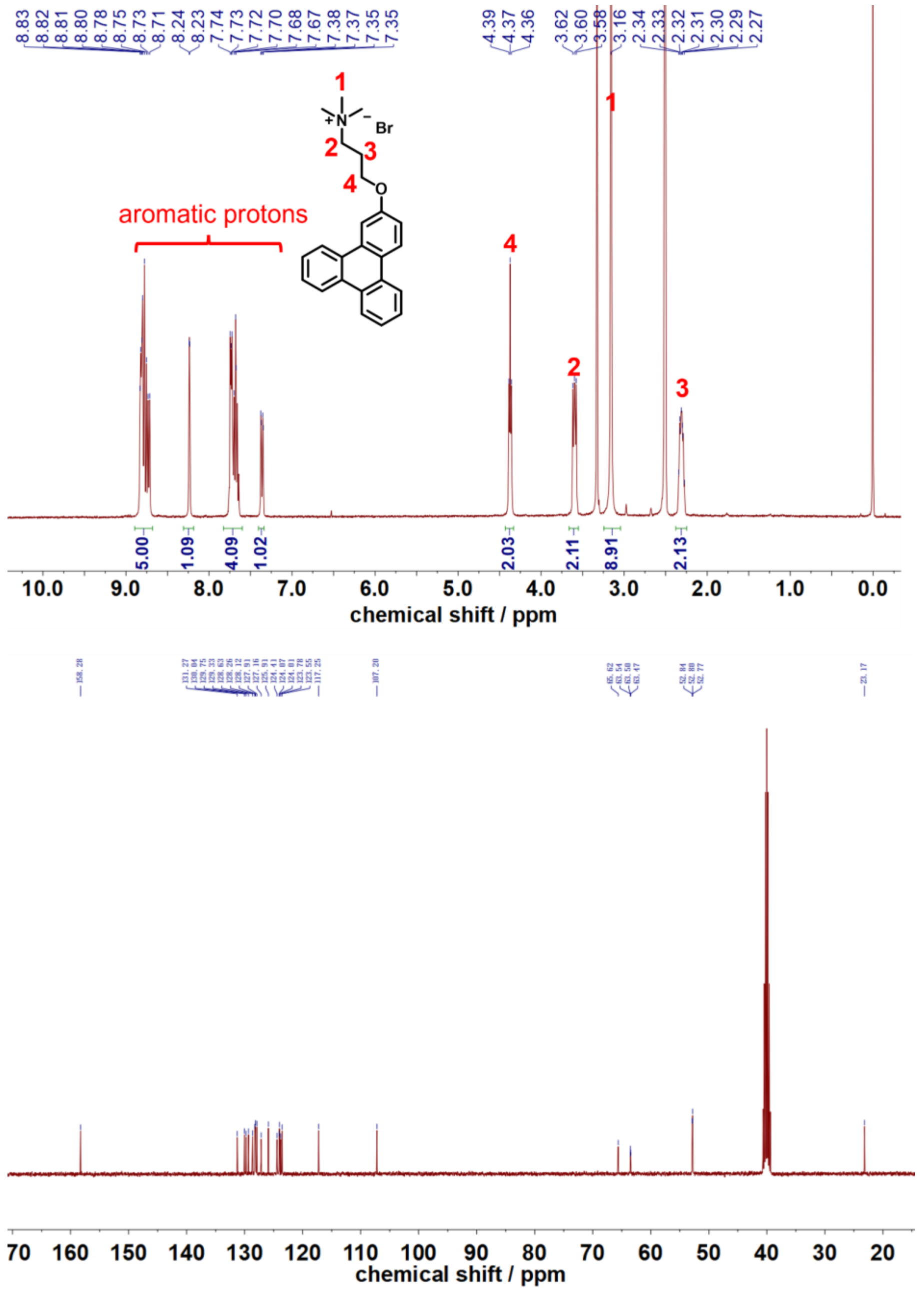

Figure S50. ^{1}H NMR spectrum and ^{13}C NMR spectrum (100 MHz, DMSO-d_6, 298 K) of compound **TPY**.
